# The impact of demographic changes, exogenous boosting and new vaccination policies on varicella and herpes zoster in Italy: a modelling and cost-effectiveness study

**DOI:** 10.1186/s12916-018-1094-7

**Published:** 2018-07-17

**Authors:** Alessia Melegaro, Valentina Marziano, Emanuele Del Fava, Piero Poletti, Marcello Tirani, Caterina Rizzo, Stefano Merler

**Affiliations:** 1 Department of Social and Political Sciences, Bocconi University, Milano, Italy; 2 Carlo F. Dondena Centre for Research on Social Dynamics and Public Policy, Bocconi University, Milano, Italy; 3 Center for Information and Communication Technology, Bruno Kessler Foundation, Trento, Italy; 4 Department of Hygiene and Preventive Medicine, ATS, Bergamo, Italy; 5 Department of Infectious Disease, Istituto Superiore di Sanità, Roma, Italy

**Keywords:** Varicella, Chickenpox, Herpes zoster, Shingles, Vaccination, Italy, Cost-effectiveness, Modelling, Individual-based models, Demography, Immunisation

## Abstract

**Background:**

The present study aims to evaluate the cost-effectiveness of the newly introduced varicella and herpes zoster (HZ) vaccination programmes in Italy. The appropriateness of the introduction of the varicella vaccine is highly debated because of concerns about the consequences on HZ epidemiology and the expected increase in the number of severe cases in case of suboptimal coverage levels.

**Methods:**

We performed a cost-utility analysis based on a stochastic individual-based model that considers realistic demographic processes and two different underlying mechanisms of exogenous boosting (temporary and progressive immunity). Routine varicella vaccination is given with a two-dose schedule (15 months, 5–6 years). The HZ vaccine is offered to the elderly (65 years), either alone or in combination with an initial catch-up campaign (66–75 years). The main outcome measures are averted cases and deaths, costs per quality-adjusted life years gained, incremental cost-effectiveness ratios, and net monetary benefits associated with the different vaccination policies.

**Results:**

Demographic processes have contributed to shaping varicella and HZ epidemiology over the years, decreasing varicella circulation and increasing the incidence of HZ. The recent introduction of varicella vaccination in Italy is expected to produce an enduring reduction in varicella incidence and, indirectly, a further increase of HZ incidence in the first decades, followed by a significant reduction in the long term. However, the concurrent introduction of routine HZ vaccination at 65 years of age is expected to mitigate this increase and, in the longer run, to reduce HZ burden to its minimum. From an economic perspective, all the considered policies are cost-effective, with the exception of varicella vaccination alone when considering a time horizon of 50 years. These results are robust to parameter uncertainties, to the two different hypotheses on the mechanism driving exogenous boosting, and to different demographic projection scenarios.

**Conclusions:**

The recent introduction of a combined varicella and HZ vaccination programme in Italy will produce significant reductions in the burden of both diseases and is found to be a cost-effective policy. This programme will counterbalance the increasing trend of zoster incidence purely due to demographic processes.

**Electronic supplementary material:**

The online version of this article (10.1186/s12916-018-1094-7) contains supplementary material, which is available to authorized users.

## Background

Varicella zoster virus (VZV) is a DNA virus belonging to the *Herpesviridae* family that affects only humans. Infection by VZV can result in two distinct diseases: varicella or chickenpox, which is a highly communicable and widespread childhood disease, and herpes zoster (HZ) or shingles, caused by the reactivation of VZV, which remains latent in the dorsal root ganglia after primary varicella infection. Although it is usually a mild disease with a relatively low percentage of complications, especially when occurring in immunocompetent children, varicella is highly contagious and may lead to more severe consequences and disabling symptoms in adults. Reactivation of the virus, usually occurring in the elderly or immunocompromised patients, leads to HZ infection. This is characterised by a vesicular eruption along the course of the nerve and is commonly associated with pain. Complications of HZ occur in up to 20% of the cases among those aged 50 or older, with post-herpetic neuralgia (PHN) being the most common, persistent, and intractable chronic sequela [1, 2].

A live attenuated vaccine against varicella was developed in 1974 and introduced in some countries starting from 1995 [3]. In Italy, eight regions (Apulia, Basilicata, Calabria, Sardinia, Sicily, Tuscany, Veneto, and Friuli-Venezia Giulia) have gradually introduced childhood varicella vaccine into their immunisation programmes starting in 2003, in children aged 13–15 months and 5–6 years [4]. Since 2017, a two-dose schedule has been introduced nationally for all newborns as one of the ten vaccines (hexavalent, plus measles, mumps, rubella, and varicella) that have become compulsory for school attendance [5]. Nonetheless, in many developed countries, the introduction of varicella vaccination into the national schedule still represents an ongoing open discussion. Indeed, VZV mass immunisation would reduce varicella circulation, but it may potentially increase the incidence of more severe varicella cases among adults [6] and reduce the partial protection against HZ provided by VZV re-exposure (called “exogenous boosting”), thus increasing HZ incidence [7]. Results from HZ surveillance programmes in countries that have introduced VZV mass immunisation do not provide univocal evidence. Some countries detected an increase in HZ incidence following mass immunisation, while others did not observe any effect on it [8, 9]. Conversely, an increase in HZ incidence has been reported in the past decades across various countries before the introduction of varicella vaccination programmes [10]. This pattern appears consistent with results from modelling work that showed that past demographic changes and, in particular, the ageing of the population may have generated the remarkable growth of HZ incidence observed in Spain between 1997 and 2004, before the introduction of the varicella vaccination programme [11].

A live attenuated vaccine against HZ was licenced in 2006 [12, 13], and so far has been recommended in some countries, either in combination with the varicella vaccination (e.g. in the USA and recently in Italy) or alone (e.g. in France and the UK), making the evaluation of post-vaccination trends even more complex.

Previous transmission models of VZV infection suggested an increase in HZ incidence as a consequence of the reduction of exogenous boosting associated with varicella vaccination. However, the magnitude of this increase depends on modelling assumptions on the mechanism of VZV reactivation [14–19], whose biology has not yet been elucidated [20–25]. The cost-effectiveness analysis here is performed under two different assumptions regarding the mechanism of VZV reactivation. The first assumption, which has been widely adopted in past modelling approaches, hypothesises temporary complete immunity to HZ following re-exposure to VZV [16]. The second assumption relies instead on the explicit modelling of the development of a progressive partial immunity to HZ following each re-exposure to VZV, which better reflects the biological mechanisms driving the exogenous boosting [7]. So far, models including the latter mechanism have provided a better fit to the age-specific profile of HZ incidence in several countries [26], Italy included [18].

Also, the cost-effectiveness of varicella vaccination programmes was shown to be highly dependent on the assumptions about the boosting mechanism [27, 28]. Varicella vaccination appeared cost-effective when the model excluded the effect of boosting on the epidemiology of HZ [27, 29]. Conversely, when the latter effect was included, the cost-effectiveness of the programme became questionable, due to detrimental effects of varicella vaccination on HZ in the short and the medium term [30, 31].

The aim of this work is to provide a thorough evaluation of the expected effectiveness and cost-effectiveness of the recently introduced Italian varicella and HZ vaccination programme. For this purpose, a stochastic individual-based model (IBM) will be used, developed considering the observed demographic processes (such as the decline of fertility). Alternative immunisation strategies will be considered, and the sensitivity of our results to the two different assumptions about the exogenous boosting will be assessed.

## Methods

In this study, we use a stochastic IBM for VZV transmission and reactivation in Italy, informed with historical demographic data and available demographic projections [32, 33] and calibrated on the age-specific varicella serological profile and age-specific HZ incidence.

The proposed modelling approach is similar to that adopted to investigate historical epidemiological trends of measles across different countries and varicella in Spain [11, 34, 35].

The model is used to assess the economic impact of different varicella and HZ vaccination strategies on the future epidemiology of the two diseases through a cost-effectiveness analysis. Details of the demographic and epidemiological data used to parameterise the model are provided in Additional file 1: Figures S1 and S4.

We consider two epidemiological models, which differ in the assumption made to model the mechanism driving exogenous boosting and VZV reactivation [7], denoted respectively as *progressive immunity* (PI) and *temporary immunity* (TI) [16, 18]. The structures of the corresponding models are shown in Additional file 1: Figures S2 and S3. In both models, maternal antibodies confer protection against varicella infection to newborn babies for 6 months on average, after which children become susceptible to natural VZV (i.e. wild-type) infection. Susceptible individuals are exposed to a time- and age-dependent force of infection. After recovery, varicella-infected individuals acquire lifelong immunity against varicella. The generation time of varicella infection is assumed to be 3 weeks on average. In model PI, after recovery from varicella, individuals become susceptible to HZ. The rate of VZV reactivation decreases with the number of re-exposures to VZV, while it increases with both the time elapsed since the last re-exposure and the individual’s age [18]. In model TI, individuals who recover from varicella acquire temporary full protection against HZ, and the VZV reactivation rate only depends on the individual’s age [16]. In both models, HZ-susceptible individuals may either develop HZ, acquiring, after recovery, lifelong immunity to HZ disease, or they can be boosted through VZV re-exposure. In model PI, each boosting event progressively reduces the risk of VZV reactivation into HZ, whereas in model TI, it provides a temporary complete protection against HZ development. In both models, we assume that only a fraction of contacts with VZV-infected individuals results in an effective boosting event [36]. Epidemiological parameters of both models' structures are provided in the Additional file 1: Table S1 and S2.

Varicella vaccine is administered in our models starting from the year 2017. Vaccinated individuals either develop lifelong protection against varicella or they undergo vaccine failure. In the latter case, they remain susceptible to VZV and may experience a milder varicella infection, called “breakthrough varicella”. Although individuals infected with breakthrough varicella can transmit the virus, they are assumed to be half as contagious as natural varicella cases [37].

Under model PI, individuals who have recovered from breakthrough varicella become susceptible to HZ, whereas under model TI, they become temporarily immune against HZ. In both models, varicella vaccinated individuals can develop HZ, either after recovery from varicella breakthrough or directly from the vaccine strain, or can experience boosting. The VZV reactivation rate for varicella vaccinated individuals is lower than for those who experienced natural varicella [38]. Individuals successfully vaccinated against HZ acquire lifelong immunity to VZV reactivation, whereas those experiencing HZ vaccine failure remain susceptible to HZ.

Five different vaccination scenarios are considered and compared in an incremental cost-effectiveness analysis. The case of no intervention is also explored to assess the expected evolution of varicella and HZ epidemiology as driven by the changing demography only, had vaccination not been introduced. Following the new Italian National Immunisation Plan (NIP) 2017–2019 [39], we implement a routine varicella vaccination programme with a two-dose schedule (first dose at 15 months of age, second dose at 5–6 years of age) and an HZ vaccination programme with the live attenuated vaccine, targeted at individuals who are 65 years old. The two policies are evaluated either as single strategies or in combination. In addition, we also evaluate the effects of a catch-up campaign with the HZ vaccine targeting 66- to 75-year-old individuals. The resulting five programmes are the following: (1) routine varicella vaccination (V_R_), (2) routine HZ vaccination (HZ_R_), (3) routine HZ vaccination with HZ catch-up campaign (HZ_R + CU_), (4) routine varicella and HZ vaccinations (V_R_HZ_R_), and (5) routine varicella and HZ vaccinations with HZ catch-up campaign (V_R_HZ_R + CU_). Base case coverage levels for varicella and HZ vaccination are assumed to be equal to 80% and 60%, respectively. The vaccine efficacy per dose is set to 80% for the varicella vaccine, which implies an efficacy of 96% after two doses [40], while it is set to 50% for one dose of HZ vaccine [13]. The efficacy of varicella vaccine only refers to the protection against VZV infection. This means that a larger efficacy for the varicella vaccine generates in our model a larger proportion of individuals not developing varicella.

Finally, demographic changes are simulated in the period 2015–2100 as informed by temporal variations of the crude birth and age-specific mortality rates provided by the United Nations in the 2015 World Population Prospects [38].

The calibration of the models was carried out using Monte Carlo Markov chain (MCMC) methods applied to the binomial likelihood of the VZV seroprevalence profile in 1996–1997 [41] and to the Poisson likelihood of the age-specific HZ incidence in 2004 (Additional file 1: Figure S4) [2]. We calculated 95% prediction intervals (PIs) for the model-based estimates. More details are provided in Additional file 1 where the robustness of our results is assessed (Figures S5 and S6). Modelling and data analyses were conducted in C and R.

### Cost-effectiveness analysis

Cost-effectiveness analysis is applied to the outcomes of the epidemiological model, and quality-adjusted life years (QALYs) gained are used to evaluate the impact of different policies in terms of reduction of disease burden. Varicella cases are differentiated between natural infection and breakthrough cases, as the latter are expected to incur lower QALY losses and generate lower costs (Additional file 1: Figure S11). For HZ, we distinguish between cases that develop post-herpetic neuralgia (PHN) and those that do not, as costs and benefits for the two conditions are expected to differ (Additional file 1: Figure S12). We consider both the direct costs of disease (general practitioner (GP) visits, treatment, and hospitalisation) and the costs of the vaccination programmes. We report on the effects of the different policies on varicella and HZ, in terms of both burden of illness (averted cases and deaths, by disease) and economic and quality of life impact (QALY gained *∆E*, and net costs *∆C*).

Cost-effectiveness outcomes are produced under the taxpayer perspective and evaluated at three different time horizons (TH = 25, 50, 85 years), assuming discount rates of either 3% or 0% per year for both future health benefits and costs. The 3% discount rate puts less weight on the cases predicted in the long term, while the 0% discount rate weighs cases at a greater distance in time as much as those closer to the origin.

We perform an incremental cost-effectiveness analysis to determine, for each model and time horizon, which policies are deemed cost-effective, using both the incremental cost-effectiveness ratio (ICER, computed as *∆C*/*∆E*) and the net monetary benefit (NMB, computed as *t∆E* − *∆C*), where the threshold *t* represents the opportunity cost of an additional QALY gained. We consider two possible cost-effectiveness (CE) thresholds *t*, one demand-based of 40,000 EUR [42], and one supply-based of 15,000 EUR [43]. The former threshold represents the dominating approach in all health care systems, including those in Italy [42], and it depends on how individuals value health compared to other types of consumptions. The latter, based on the estimated marginal productivity of the health care system, represents a direct measure of the health consequence of changes in the allocation of the available resources [43]. A sensitivity analysis is conducted to assess how cost-effectiveness analysis results change when assuming different values of the CE threshold *t*.

The base case analysis considers 1000 model realisations of varicella and HZ cases by age and over time, generated under the strategies under investigation. These are combined with the base case values of the economic and quality of life parameters (Tables 1 and 2). The robustness of model results to uncertainty in model parameters is assessed through a probabilistic sensitivity analysis (PSA) [44], where, using an empirical Bayesian approach, the prior distributions for the model parameters are either grounded on the respective base case values or are set by assuming little or no information about the parameters of interest (e.g. when any estimate of the variability of the parameter is not available) [45]. For evaluating the effects of the uncertainty around both epidemiological estimates and economic model parameters, we compute the NMB associated with 1000 different parameter sets, sample from their posterior distribution, and derive 95% credible intervals (CIs) from their posterior distribution. The uncertainty around the choice of the optimal strategy (i.e. generating the highest NMB), under both discount rates of 0% and 3%, is represented with (1) box plots of the posterior distribution of the NMB and (2) net benefit charts showing how the median NMB changes for a variety of values of the CE threshold [46]. Finally, six additional scenarios under the PSA are evaluated to assess the sensitivity of our results to (1) two different coverage levels of varicella vaccination (70%, 95%), (2) two extreme assumptions on the role of exogenous boosting (assuming either low or high reduction of the HZ risk due to VZV re-exposure), and (3) two different demographic scenarios on the total fertility rate in the future (a lower and a higher crude birth rate).

**Table 1:** Epidemiological and quality of life (QALY) parameters of the economic model. We report the base case values and the standard deviations, taken either from the literature or from administrative data, the shapes of the prior distribution, the 95% CI from the posterior distribution of the parameters, and the source of the base case values

Parameter	Base case values	Standard deviations	Prior Distribution	95% CI Posterior distribution	Source
Epidemiological parameters					
Proportion of HZ cases developing PHN^c^	0.049 (by age)	0.0023 (by age)	Beta	–	[2]
Hosp. rate for natural varicella (NV) per model TI/PI (per 1000 cases)	2.35/2.36 (by age)	0.61/0.62 (by age)	Beta	–	Estimate^a^
Breakthrough varicella (BV) vs. NV hosp. rate	0.25	0.05	Beta	[0.16–0.35]	[58, 59]
Hosp. rate for HZ per model TI/PI (per 1000 cases)	13.60/13.12 (by age)	4.49/4.63 (by age)	Beta	–	Estimate^a^
Hosp. rate for PHN per model TI/PI (per 1000 cases)	41.55/40.77 (by age)	9.53/10.39 (by age)	Beta	–	Estimate^a^
Case fatality rate for NV (per 1000 hospitalised)	4.01 (by age)	2.98 (by age)	Beta	–	Estimate^b^
BV vs. NV case fatality rate	0.005	0.0022	Beta	[0.002–0.01]	[59, 60]
HZ-PHN case fatality rate (per 1000 hospitalised)	12.70 (by age)	5.43 (by age)	Beta	–	Estimate^b^
No. GP consultations per NV case					
< 14 years	2	0.2	Gamma	[1.63–2.43]	[59]
≥ 15 years	1	0.2	Gamma	[0.64–1.43]	[59]
No. GP consultations per BV case	0.5	0.05	Gamma	[0.41–0.60]	[59]
Quality of life measures					
Overall weighted health state index (EQ-5D_index_)	0.84 (by age)	0.21 (by age)	Beta		[61]
Weighted health state index varicella					
< 14 years	0.81	0.031	Beta	[0.76–0.86]	[31]
≥ 15 years	0.73	0.025	Beta	[0.68–0.78]	[62]
Prob. severe NV cases	0.65	0.0063	Beta	[0.64–0.66]	[63]
Prob. severe BV cases	0.25	0.011	Beta	[0.23–0.27]	[63]
Reduction in QALY loss Mild vs. severe varicella cases	0.25	0.10	Beta	[0.08–0.47]	[48]
QALY loss HZ					
20 years	0.022	0.0018	Beta	[0.019–0.026]	[48]
40 years	0.031	0.0030	Beta	[0.026–0.037]	[48]
60 years	0.064	0.0082	Beta	[0.049–0.081]	[48]
80 years	0.19	0.030	Beta	[0.14–0.25]	[48]

^a^Average number of hospitalisations by age due to varicella, HZ, and PHN (Hospital Discharge Register, 2001–2012) divided by the predicted pre-vaccination incidence generated by the epidemiological model. PHN incidence is derived by multiplying the estimated HZ incidence by the probability of HZ cases developing PHN [2]

^b^Average number of deaths by age due to varicella (Italian National Health Institute, 2001–2012) and HZ (European Union detailed mortality database, 2001–2012) divided by the respective estimates of the hospitalisation rates

^c^PHN cases lasting at least 3 months

**Table 2:** Cost of disease and vaccination parameters of the economic model. We report the base case values and the standard deviations, taken either from the literature or from administrative data, the shapes of the prior distribution, the 95% CI from the posterior distribution of the parameters, and the source of the base case values

Parameter	Base case values (EUR)	Standard deviation (EUR)	Prior Distribution	95% CI Posterior distribution	Source
Cost of disease parameters					
GP consultation cost for NV^a^					
< 14 years	29.07	2.05	Gamma	[25.14–33.27]	[64]
≥ 15 years	19.00	1.25	Gamma	[16.65–21.53]	[65]
GP treatment cost for NV^a^					
< 14 years	13.64	1.61	Gamma	[10.72–16.96]	[66]
≥ 15 years	26.10	2.00	Gamma	[22.35–30.14]	[65]
Hospitalisation cost for NV					
< 14 years	2683.25	1780.56	Gamma	[411.80–7128.02]	Hospital Discharge Register (HDR) Lombardy
≥ 15 years	2720.29	2573.64	Gamma	[348.73–9463.01]	HDR Lombardy
Outpatient cost for HZ (incl. Visit, treatment, and diagnostics)^a^	144.03	114.48	Gamma	[11.53–438.21]	[2]
Outpatient cost for PHN (incl. Visit, treatment, and diagnostics)^a^	523.72	520.05	Gamma	[13.95–1905.90]	[2]
Hospitalisation cost for HZ					
< 49 years	2073.50	2260.35	Gamma	[176.35–8238.92]	HDR Lombardy
≥50 years	2020.23	1332.60	Gamma	[307.63–5313.47]	HDR Lombardy
Hospitalisation cost for PHN					
< 49 years	1500.74	1714.41	Gamma	[78.93–6132.75]	HDR Lombardy
≥50 years	1927.25	1892.80	Gamma	[75.02–7058.80]	
Vaccination parameters					
Cost per dose of varicella vaccination	31.46		Fixed	–	Purchase price^b^
Cost per dose of HZ vaccination	87.00		Fixed	–	Invitation for bid
Admin. cost per dose of vaccination^a^	7.56		Fixed	–	[59]

^a^These costs are adjusted for the inflation at 2015, using the Italian Consumer Price Index

^b^Purchase price of the vaccine for varicella per dose, paid by Lombardy Regional Health System

## Results

**Fig. 1:**
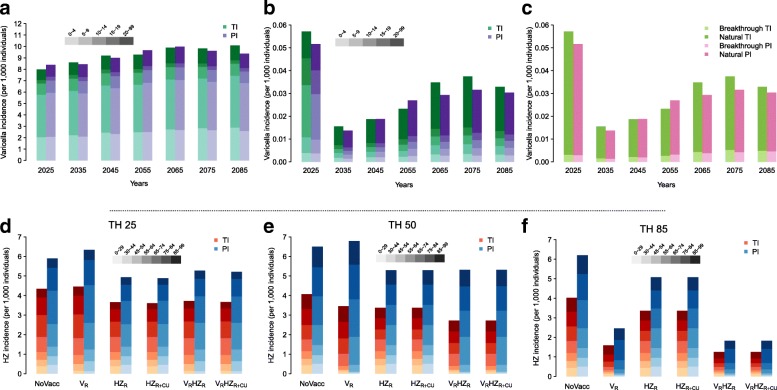
Estimated total varicella incidence over time, stratified by age groups, as obtained by models TI and PI, under (**a**) the no vaccination scenario and (**b**) the combined varicella and HZ routine vaccinations V_R_HZ_R_. **c** Estimated total varicella incidence over time, stratified by type of infection (natural vs. breakthrough varicella), as obtained by models TI and PI, under the combined varicella and HZ routine vaccinations V_R_HZ_R_. Estimated total HZ incidence, stratified by age groups, as obtained by models TI and PI, under the different considered policies at a time horizon of **d** 25 years, **e** 50 years, and **f** 85 years

**Fig. 2:**
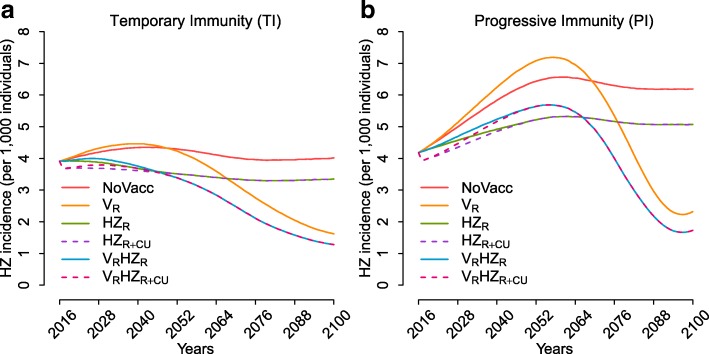
Estimated total HZ incidence over time as obtained under the different vaccination strategies, for model TI (**a**) and model PI (**b**)

Under the no vaccination scenario, both model structures predict a stable overall incidence of varicella over time (Fig. 1a), as well as an increase in HZ rate, which will stabilise only after some decades (Fig. 1d–f and Fig. 2). This growth can be ascribed to two factors, i.e. the population ageing that acts equally in both models, and the delayed effect, stronger for model PI, of the decline in the fertility rate which occurred during the last century on the individual risk of HZ development. Indeed, the decline of fertility in the past reduced both varicella circulation and the frequency of VZV re-exposure. In particular, during the period 2017–2100, model TI forecasts a peak in the total HZ incidence of about 15.1% (95% PI 8.1–22.3%) with respect to 2017 and a stable incidence level in the long term that is 2.6% (95% PI –5.2 to 10.9%) higher than in 2017. On the other hand, model PI forecasts a peak in HZ incidence with respect to 2017 that amounts to 61.4% (95% PI 44.9–77.1%) and a stable incidence level in the long term that is 48.3% (95% PI 33.4–62.8%) higher than in 2017 (Fig. 2).

The recently introduced combined varicella and zoster vaccination strategy (V_R_HZ_R_), at baseline coverage levels of 80% and 60%, respectively, is expected to produce a sudden and enduring reduction in varicella incidence as well as a significant increase in the average age at varicella infection (Fig. 1b). Moreover, although in the short and the medium term the current programme might provide an increase in HZ incidence compared to the pre-vaccination level (though still lower than under the no vaccination scenario), in the long term it is expected to reduce the burden of HZ disease in Italy to its minimum, with small differences between the two models (Fig. 1d–f and Fig. 2, Additional file 1: Figures S9 and S10). In particular, we expect a reduction of 70.7% (95% PI 34.7–91.5%) in HZ incidence with respect to the no vaccination scenario under model PI, and of 68.6% (95% PI 61.8–74.1%) under model TI (Fig. 1f), with an upwards shift in the average age at VZV reactivation into HZ. Indeed, for both models, while the age group that is mostly affected by HZ in 2017 is 55–64 years (accounting on average for 21.1% and 20.3% of the total cases in models PI and TI, respectively), in the long term the models forecast that most cases would occur in the age group 75–84 under model PI (40% on average) and in the age group 85–99 under model TI (26.8% on average). Nonetheless, the overall reduction in HZ incidence obtained with the combined programme (V_R_HZ_R_) implies a much lower cumulative number of HZ cases and HZ-related deaths than those expected with no vaccination, under any time horizon (Additional file 1: Tables S3 and S4, with 3% and 0% discount rates, respectively).

The effect of the introduction of a varicella vaccination policy alone (V_R_) on natural HZ incidence in the short and the medium term strongly depends on the model considered. In the first decades after introduction, no evident variation with respect to the no vaccination scenario is expected under model TI, whereas a 15% increase of HZ incidence is estimated under model PI (Fig. 2). In the long term, since varicella vaccination would reduce the replacement of the HZ-susceptible individuals generated by natural varicella, we would find that HZ incidence is less than half of that expected under no vaccination (Fig. 1f), with levels even lower than those in the pre-vaccination period (Fig. 2). Conversely, a routine HZ vaccination programme would mitigate the increase of HZ incidence both in the absence (HZ_R_) and in the presence of varicella vaccination (V_R_HZ_R_), in both the short and the medium term. However, the policy including only HZ vaccination would not affect the replacement of the HZ-susceptible individuals caused by varicella infection, and therefore result in the long term in a much higher level of HZ incidence than that achieved through policy V_R_ (Fig. 1f). Indeed, according to model PI, in the long term, V_R_ and HZ_R_ would respectively lead to a 60.6% (95% PI 12.6–88.2%) and an 18.1% (95% PI 15.9%–20.4%) reduction in HZ incidence with respect to no vaccination (although the undiscounted cumulative number of zoster cases remains slightly higher under policy V_R_ (Additional file 1: Table S4).

Under both models and time horizons (except for TH = 50 under the PI model) and assuming a discount rate of 3%, we found the combined policy with varicella vaccination and HZ vaccination with catch-up (V_R_HZ_R + CU_) to be the most cost-effective in terms of ICER and NMB, no matter what the chosen threshold (Table 3). However, for TH = 50 years and when considering model PI, the most cost-effective policy remains the one with the HZ vaccination and catch-up campaign (HZ_R + CU_) (Table 3).

**Table 3:** Cost-utility analysis for the different vaccination policies, by model and by time horizon

Model TI						Model PI					
Policy	Total cost^a^	QALY loss^b^	ICER^c^	NMB_k_^d^	NMB_v_^e^	Policy	Total cost^a^	QALY loss^b^	ICER^c^	NMB_k_^d^	NMB_v_^e^
Time horizon = 25 years											
No vacc.	1812	455	–	–	–	No vacc.	2023	538	–	–	–
V_R_	1831	446	2219	110	324	V_R_	2066	537	WD	−37	−28
HZ_R_	2540	413	WD	−94	963	HZ_R_	2722	481	WD	143	1548
V_R_HZ_R_	2555	402	WD	50	1371	V_R_HZ_R_	2757	477	WD	166	1667
HZ_R + CU_	2823	380	WD	109	1975	HZ_R + CU_	2995	444	WD	436	2782
V_R_HZ_R + CU_	2836	369	12,989	265	2414	V_R_HZ_R + CU_	3028	439	10,175	476	2944
Time horizon = 50 years											
No vacc.	2668	691	–	–	–	No vacc.	3145	885	–	–	–
V_R_	2643	680	Cost-saving	190	466	V_R_	3240	924	SD	−679	−1654
V_R_HZ_R_	3563	576	WD	820	3679	HZ_R_	3989	745	6031	1255	4754
HZ_R_	3601	593	SD	531	2970	V_R_HZ_R_	4047	767	SD	878	3844
V_R_HZ_R + CU_	3841	543	8722	1052	4760	HZ_R + CU_	4258	707	6984	1563	6022
HZ_R + CU_	3880	560	SD	749	4017	V_R_HZ_R + CU_	4313	727	SD	1204	5156
Time horizon = 85 years											
No vacc.	3092	798	–	–	–	No vacc.	3724	1059	–	–	–
V_R_	2962	759	Cost-saving	717	1695	V_R_	3704	1072	1517	−174	−497
V_R_HZ_R_	3984	634	8170	1570	5674	V_R_HZ_R_	4572	874	4375	1934	6571
HZ_R_	4119	676	SD	805	3859	HZ_R_	4627	878	SD	1824	6367
V_R_HZ_R + CU_	4264	600	8266	1799	6752	V_R_HZ_R + CU_	4842	834	6829	2257	7881
HZ_R + CU_	4402	643	SD	1020	4904	HZ_R + CU_	4898	839	SD	2128	7632

All outcomes are reported with a 3% discount rate for both benefits and costs

^a^Accounting for cost of disease and cost of policy, in million EUR

^b^In thousands

^c^*SD* strong dominance (a policy is dominated when the alternative is less costly and more effective), *WD* weak dominance (a policy is dominated when its ICER is larger than that of a policy with higher effectiveness). The ICER is measured in EUR/QALY gained

^d^Based on the marginal productivity of the national health system (*t* = 15,000 EUR) and calculated with respect to no vaccination, in million EUR

^e^Based on the consumers’ willingness to pay (*t* = 40,000 EUR) and calculated with respect to no vaccination, in million EUR

Varicella vaccination alone (V_R_) was never found to perform better than the other strategies, even though it strongly dominated the no vaccination scenario under model TI, resulting in cost savings in the medium and in the long term (Table 3). Conversely, V_R_ performed worse under model PI, where it turned out to be always dominated and even generated QALY losses in the medium and in the long term as a consequence of the increase in cumulative HZ cases in the first decades following the introduction of vaccination (Additional file 1: Table S3). Similar conclusions can be drawn when considering a discount rate of 0% (Additional file 1: Table S4). Also with undiscounted values, we found the combined policy V_R_HZ_R + CU_ to be the most cost-effective for all models and time horizons, except for TH = 50 under model TI, where the policy HZ_R + CU_ was the most cost-effective (Additional file 1: Table S5).

**Fig. 3:**
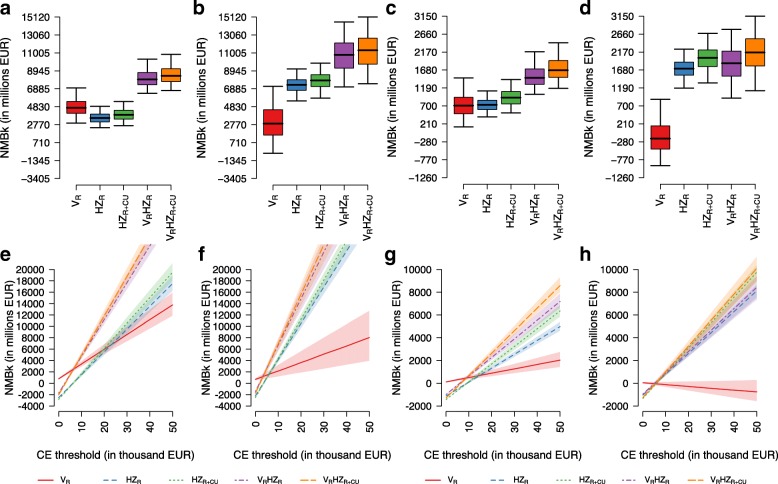
Box plots of the posterior distribution of the NMB (with 2.5%, 25%, 50%, 75%, and 97.5% quantiles) obtained, under the PSA, from the comparison between each policy and the no intervention scenario, using the conservative supply-based threshold *t* of 15,000 EUR, under model TI (**a** and **c**) and model PI (**b** and **d**), and under the longest time horizon (85 years). Discount rates for both costs and benefits are assumed to be equal to 0% (**a** and **b**) and to 3% (**c** and **d**). Net benefit charts of the NMB obtained under different CE thresholds and under the longest time horizon (85 years), for **e** model TI and 0% discount rate; **f** model PI and 0% discount rate; **g** model TI and 3% discount rate; **h** model PI and 3% discount rate

Under the PSA, we found that, for a CE threshold of 15,000 EUR, V_R_ consistently underperforms compared to the other strategies, irrespective of the model used and the assumed discount rate. Its worst performance, with the NMB decreasing as the CE threshold increases, is expected under model PI with discount rate equal to 3%, because of the higher (negative) impact of varicella vaccination on HZ epidemiology (Fig. 3h). On the contrary, under model TI, V_R_ always generates a strictly positive NMB, but it is always dominated by the other strategies, except for CE thresholds lower than 8000 EUR (Fig. 3g).

Under both models, the combination of the two vaccinations, V_R_HZ_R_ and V_R_HZ_R + CU_, maximises the NMBs when considering undiscounted outcomes (Fig. 3a and b), with the latter strategy being the most cost-effective above a CE threshold of 8000 EUR (Fig. 3e and f). However, when assuming a 3% discount rate, we find that, under model PI, the reductions in HZ incidence in the long term are not enough to counterbalance the short-term increase in HZ infections induced by varicella vaccination. Hence, the resulting NMB distribution of the combined programmes is quite similar to those generated by HZ_R_ and HZ_R + CU_ (Fig. 3d and h). In particular, increasing the CE threshold, the strategy V_R_HZ_R + CU_ converges to a probability of about 60% of being the most cost-effective, while the strategy HZ_R + CU_ converges to about 40% (Additional file 1: Figure S14D). This result shows that the introduction of an HZ catch-up programme for those aged 66–75 is always beneficial, as it usually produces an increase in the estimated NMB.

Interestingly, we found that the input parameters for the epidemiological model, rather than those for the economic model, represented the most influential source of uncertainty on the posterior distribution of the NMB (Additional file 1: Figure S26 and Table S6).

Remarkably, the obtained results are quite robust to variations in the varicella vaccination coverage levels, although upwards or downwards variations in the coverage generate, respectively, a decrease or an increase in the estimated NMBs (Additional file 1: Figures S15 and S16). When we assume a weaker boosting effect, i.e. a low reduction of the HZ risk due to VZV re-exposure, the distribution of the NMBs for the single HZ policies shifts downwards, and the combined policies V_R_HZ_R + CU_ and V_R_HZ_R_ outperform the single ones (Additional file 1: Figure S17). Conversely, by considering a stronger role of boosting, both combined policies (V_R_HZ_R_ and V_R_HZ_R + CU_) underperform compared to those solely based on HZ vaccination (HZ_R_ and HZ_R + CU_), while V_R_ never proves cost-effective, even in the long term (Additional file 1: Figure S18). Considering the uncertainty in future demographic changes, scenarios assuming a higher future birth rate result in a higher varicella and a lower HZ incidence. However, under a high birth rate scenario, the population would also increase, leading to a higher number of HZ cases, despite the drop in the incidence. Nevertheless, in the long term, the results of the cost-effectiveness analysis are robust to the uncertainty in the demography, with the combined strategies generating the highest NMBs under both the low and the high birth rate scenarios (Additional file 1: Figures S23 and S24).

## Discussion

### Principal findings

The results of this study suggest that the recent introduction of the combined varicella and HZ vaccination programmes in Italy is expected to produce a significant reduction of the disease burden caused by VZV infection and reactivation in the long term. The new policy appears to be economically acceptable from a public health payer perspective under different model assumptions on the mechanism of exogenous boosting. In the base case analysis, under the progressive immunity (PI) model, we found that the combined programme would annually prevent, with respect to no vaccination, an average of 435,000 undiscounted cases of varicella, more than 77,000 cases of HZ, and 81 HZ-related deaths per year at a cost of 4375 EUR per QALY gained. Instead, under the temporary immunity (TI) model, we would predict a reduction of almost 437,000 varicella cases, 59,000 HZ cases, and 45 HZ-related deaths per year at a cost of 8170 EUR per QALY gained. From our modelling results, the projected impact would be further improved by adding an HZ catch-up campaign targeting persons 66–75 years old to the implemented national programme. The inclusion of this targeted immunisation activity could prevent on average 3542 additional cases of HZ and 6 deaths per year under model PI, or 3079 HZ cases and 5 deaths under model TI, always maximising the estimated NMB of the strategy. The NMBs are mostly sensitive to the uncertainty on epidemiological parameters, and the ICER values strongly depend on parameters related to HZ and PHN, such as the outpatient and inpatient costs and the corresponding QALY loss (Additional file 1: Figure S25). Overall results appeared robust to changes in model parameters and to different assumptions on exogenous boosting. However, when considering a post-vaccination time horizon of 25 years, varicella vaccination produced an increase in HZ incidence, the magnitude of which depended on whether we assumed a *progressive* or a *temporary* immunity model.

Individual strategies were also evaluated, and model results showed that, whereas HZ vaccination is expected to cost-effectively reduce the burden of HZ disease, varicella vaccination would negatively impact the overall burden of VZV in the short and the medium term. Hence, the introduction of this strategy on its own would not be considered cost-effective from the health care payer perspective.

### Strengths and limitations

The obtained results are relevant as they thoroughly evaluate the impact of these newly introduced vaccination programmes, taking into consideration all potential direct and indirect, both positive and negative, effects of the vaccines in Italy, exploring the current uncertainty on the mechanism, either temporary or progressive, underlying exogenous boosting. This latter aspect differentiates this study from previous work, where the cost-effectiveness was evaluated either considering no exogenous boosting or assuming a TI mechanism. In the former case, findings showed that varicella vaccination was always cost-effective (under the health payer perspective) or even cost-saving (under the societal perspective) [27, 29], while in the latter case, it was generally not cost-effective [30, 31] (except for France [47]), except when considering the long term [29, 48]. Moreover, the model also improves on the previous analyses in the way demographic processes are accommodated. Indeed, the model explicitly considers realistic changes in the Italian population age structure over time to take into account temporal trends in VZV epidemiology that are not directly ascribable to the immunisation programme, but rather to the ageing of the population and to the reduction in the expected number of susceptible children. A similar approach was also recently considered for modelling varicella and HZ epidemiology in Germany [49]. In their study, the authors evaluated the effect of population ageing and migration flows on the epidemiology of the two diseases, along with vaccination policies, but they did not assess the cost-effectiveness and economic acceptability of the programmes.

Our work is based on the underlying assumption of long-lasting protection induced by the HZ vaccine, which might appear to contrast with recent evidence of declining effectiveness (from 60 to 70% in the first years to 30 to 40% in the eighth year) [50]. Although we acknowledge that this can be considered a limitation of our work, we believe that our assumption of a constant vaccine efficacy of 50% can be seen as an average level of protection throughout the observation period. To address the issue of waning vaccine-induced immunity, a second dose of vaccine has been suggested [51]. We took into consideration this possibility by doubling the price of the considered live attenuated HZ vaccine to mimic a two-dose HZ vaccination policy. We found that HZ vaccination would still be cost-effective in the long run, even under the lower threshold of the ICER. Clearly, more work is needed in this direction, also in the light of the fact that some countries are currently considering replacing the live attenuated HZ vaccine with a new recombinant one [52, 53]. This new vaccine has recently been licenced by the US Food and Drug Administration and recommended for healthy adults aged 50 and above to prevent shingles and related complications [54]. Although its reported high efficacy is expected to enhance the cost-effectiveness of the HZ vaccination, both alone and in combination with the varicella vaccine [55], the two-dose schedule and the high price might counterbalance the positive effects.

Finally, our model assumed that the varicella vaccination was first administered to all newborn babies in Italy beginning in 2017, despite the vaccination having gradually been available in some regions since 2003. Considering that the coverage in these regions has reached moderate or high levels in recent years [56, 57], our results might underestimate the impact of varicella vaccination on VZV circulation in Italy and consequently overestimate varicella and HZ incidence, mostly in the short term [4].

### Implications for policy makers

Our findings are especially relevant when considering the very recent changes in the Italian National Immunisation Plan (NIP) 2017–2019. The NIP 2017–2019 has introduced recommendations for six new additional vaccinations, which will be administered free of charge: four vaccinations targeted to infants and children (i.e. vaccines against rotavirus, varicella, group B meningococcal disease, and human papillomavirus for boys), and two to the elderly (i.e. vaccines against pneumococcal disease and herpes zoster). These recommendations have sparked a lively and heated debate on the appropriateness of the new childhood programmes and on the fact that varicella is one of the ten vaccines (hexavalent, plus measles, mumps, rubella, and varicella) that have been introduced as compulsory for school attendance (only starting from those born in 2017). So far, Italy has shown high regional heterogeneity regarding immunisation schedules and outcomes; thus, a new structured national plan will promise a better harmonisation in the vaccine offer and uptake. At the same time, the NIP requirements for school entry should ensure the achievement and maintenance of the high vaccination coverage rates that are necessary for the desired herd immunity effects.

## Conclusions

Our study has shown that the newly introduced combined varicella and HZ vaccination strategy in Italy is expected to be effective and cost-effective in reducing the burden of disease and the loss of quality of life. Moreover, the programme is expected to counterbalance the increasing trend in HZ incidence that is estimated in the absence of any vaccination programme and is thus purely due to demographic change. In particular, under the more realistic assumption of PI for the exogenous boosting, this decline would amount to around 435,000 undiscounted cases of varicella and more than 77,000 cases of HZ (and 81 HZ-related deaths) per year at a cost of 4375 EUR per QALY gained. We also found that an additional catch-up campaign for HZ vaccination targeting people aged 66–75 would further increase the benefits of the combined programme, leading to an additional reduction of 3542 cases of HZ and 6 HZ-related deaths per year, at a cost of 6829 EUR per QALY gained.

Our work shows the importance of using models with non-stationary populations, in particular accounting for changing demography, when assessing the impact of vaccination policies on the epidemiology of those infectious diseases that are highly dependent on people mixing. This can surely provide a more thorough understanding of the expected outcomes and therefore help policy makers to design effective preventive strategies.

## Additional file

Additional file 1: Supplementary methods and results. (DOCX 6670 kb)

## Abbreviations

CE: Cost-effectiveness
CI: Credible interval
GP: General practitioner
HZ: Herpes zoster
IBM: Individual-based model
ICER: Incremental cost-effectiveness ratio
MCMC: Monte Carlo Markov chain
NIP: National Immunisation Plan
NMB: Net monetary benefit
PHN: Post-herpetic neuralgia
PI: Progressive immunity
PSA: Probabilistic sensitivity analysis
QALY: Quality-adjusted life year
TH: Time horizon
TI: Temporary immunity
VZV: Varicella zoster virus
