# Supplementary material for: The impact of demographic changes, exogenous boosting and new vaccination policies on varicella and herpes zoster in Italy: a modelling and cost-effectiveness study
Source: BMC Med. 2018 Jul 17;16:117. doi: 10.1186/s12916-018-1094-7 (PMC6048801; doi:10.1186/s12916-018-1094-7)
Supplement: Supplementary file 1 — Supplementary methods and results. (DOCX 6670 kb) [file 12916_2018_1094_MOESM1_ESM.docx]

**Additional file 1**

**The impact of demographic changes, exogenous boosting and new vaccination policies on varicella and herpes zoster in Italy: a modelling and cost-effectiveness study**

Alessia Melegaro, Valentina Marziano, Emanuele Del Fava, Piero Poletti, Marcello Tirani, Caterina Rizzo, Stefano Merler

**Table of Contents**

1. Demographic and epidemiological data ………………………………………………………2

2. Structures of the epidemiological models ……………………………………………………..3

3. Model calibration ……………………………………………………………………………...7

4. Robustness of model’s projections ……....…………………………………………………..10

5. Past VZV epidemiology in Italy (1900-2015) ……………………………………………….12

6. Future VZV epidemiology in Italy (2015-2100) ………………………………………….…13

7. Cost-effectiveness analysis …………………………………………………………………..14

8. Probabilistic Sensitivity Analysis ……………………………………………………………20

9. Assessing the impact of the different sources of uncertainties ………………………………28

10. References ……………………………………………………………………………………31

### Demographic and epidemiological data

Demographic changes occurred in Italy between 1900 and 2015 were modelled using data on yearly variations of the crude birth rate, the age-specific mortality rates, and the net migration flows as reported for this country and the considered period of time (Fig S1) [1,2]. Data on the age distribution of the Italian population during the period 1900-2015 [3] were used to validate changes in the population age structure simulated by our models (Fig S1).

Demographic changes during the prediction period (2016-2100) are based on projections on the crude birth rate available from the 2015 Revision of the World Population prospects [3]. Specifically, the main analysis performed in this manuscript uses the “medium fertility” birth rate projections (red in Figure S1), while “low” and “high” fertility projections (yellow and green in Figure S1, respectively) are used to investigate the sensitivity of our results to uncertainty surrounding future demographic changes. Results obtained in this sensitivity analysis are discussed in Section 8.2.3.

Mixing patterns by age are simulated by using the synthetic social contact matrices recently estimated for Italy [4]. Unknown epidemiological parameters of the considered transmission models were estimated using data on the Italian VZV seroprevalence profile observed in 1996-1997 [5], and on the age-specific HZ incidence in 2004 [6].


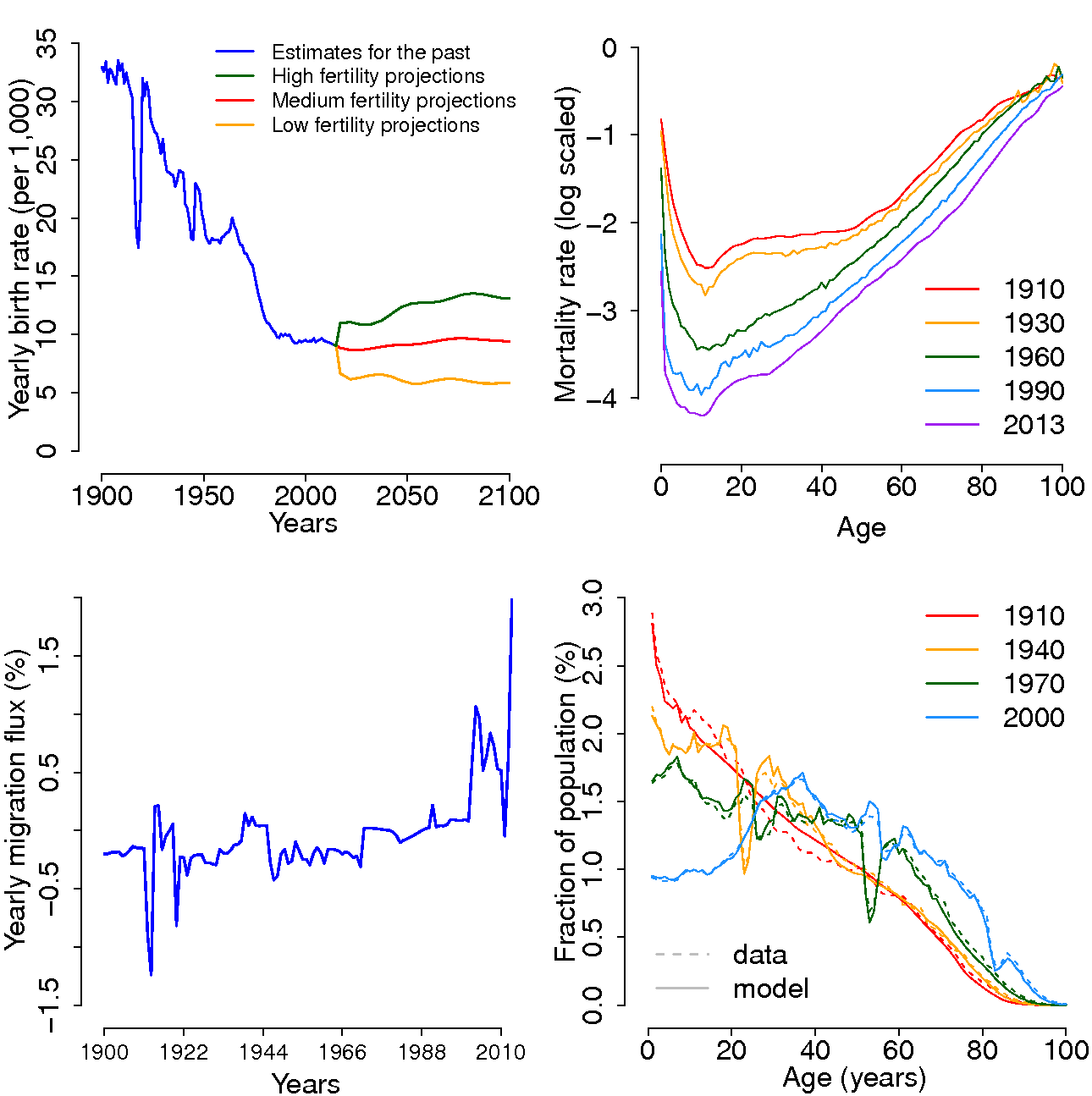


**Figure S1 Demographic changes in Italy.** From top left to bottom left, clockwise, we report the crude birth rate (per 1,000 individuals) as observed in Italy during the period 1900-2015 (blue) and as reported in the 2015 UN World Population Prospects under different demographic projections (2016-2100); the observed age-specific mortality rate (on a logarithmic scale) in different decades, the age distribution of the Italian population in different years (solid lines represent data; dotted lines represent model estimates), and the observed yearly migration flux (in percentage) over 1900-2015.

### Structures of the epidemiological models

### *Demographic transitions*

### The models used in this work are stochastic individual-based models, accounting for the vital dynamics of the Italian population as driven by births, deaths, migration fluxes, and the aging of the population occurring over time. Demographic transitions are simulated in the same way in the two considered models (temporary immunity and progressive immunity). Briefly, the population is stratified into 101 one-year age classes, *a* ∈ {0, 1, 2, ..., 99, 100+}. Each week new individuals are added to the population according to the observed crude birth rate, while, at the end of each year, individuals die according to the observed age-specific mortality rates, migrate according to the observed migration fluxes, and the age of the population is incremented by one.

### *Epidemiological transitions*

### The two models considered in this work, denoted as “progressive immunity” (model PI) and “temporary immunity” (model TI), differ in the assumptions made to model the mechanism driving exogenous boosting and VZV reactivation. Epidemiological transitions assumed for model PI and TI are summarized by the schematic diagrams shown in Fig S2 and S3, and are described in sub-sections 2.2.1 and 2.2.2.

### The model based on the progressive immunity mechanism (model PI) mimics more accurately the original biological hypothesis proposed by Hope Simpson [7], on which both models are based. Model PI has also already proven to better fit the data [8,9]. However, mechanisms driving VZV reactivation into HZ and exogenous boosting are both still poorly understood. As a consequence, goodness-of-fit may not be sufficient to determine which mechanism is more realistic. In addition, model TI has widely been used in past modelling works [10-12], therefore representing an important reference to assess the robustness of results provided by a cost-effectiveness analysis to the existing uncertainty in the mechanism of VZV reactivation.

### Model PI

### Model PI was already used in published work focusing on the impact of changing demography on the epidemiology of varicella and HZ in Spain [13].

### In the model, all individuals are assumed to be protected at birth against varicella infection by the passive transfer of maternal antibodies (M). After an average time $\frac{\boldsymbol{1}}{\boldsymbol{m}}\boldsymbol{=6}$ months, maternal immunity against VZV infection wanes and individuals become susceptible to varicella (S). Individuals vaccinated against varicella become either lifelong protected against varicella (VP_1_) or undergo vaccine failure and thus remain susceptible to breakthrough varicella (S^*^). More specifically, individuals are vaccinated against varicella at certain coverage level *c*, which is assumed to be constant over time; however, only a fraction *p* of vaccinated individuals becomes successfully immunized against VZV infection. This means that, in our model, for larger values of *p*, the varicella vaccine generates a lower proportion of individuals at risk of developing varicella. The impact of vaccination on disease severity is only indirect. In particular, vaccinated people that were not successfully immunized against VZV (a fraction *1-p* of those vaccinated) can be infected by VZV but if infected by VZV they develop milder disease symptoms (breakthrough varicella). In addition, changes in the age at infection led by sub-optimal vaccination coverage levels can change the number of severe cases as a result of the higher hospitalisation risks at older ages and higher QALY loss for both varicella and zoster.

### Varicella susceptible individuals (S and S^*^) are exposed to a time-dependent, age-specific VZV force of infection (FOI), namely,

### $\boldsymbol{\lambda}\left( \boldsymbol{a,t} \right)\boldsymbol{=\beta}\sum_{\boldsymbol{a}^{\boldsymbol{'}}} \boldsymbol{C}_{\boldsymbol{a}\boldsymbol{a}^{\boldsymbol{'}}}\left( \boldsymbol{t} \right)\boldsymbol{[y}_{\boldsymbol{a}^{\boldsymbol{'}}}\left( \boldsymbol{t} \right)\boldsymbol{+\alpha}\boldsymbol{y}_{\boldsymbol{a}^{\boldsymbol{'}}}^{\boldsymbol{*}}\left( \boldsymbol{t} \right)\boldsymbol{]}$(1)

### where

- *a*, *a’* denote individuals’ chronological age
- *t* represents time
- $C_{aa^{'}}\left( t \right)$is the average daily number of contacts that an individual of age $a$ has with individuals of age $a^{'}$ at time $t$.
- $\beta$ is an age-independent scale factor shaping the age-specific VZV transmission rate
- $y_{a^{'}}\left( t \right)$ is the fraction of individuals infected with natural varicella within the age group $a^{'}$ at time *t.*
- $y_{a^{'}}^{*}\left( t \right)$is the fraction of individuals infected with breakthrough varicella within the age group $a^{'}$ at time *t;* breakthrough varicella cases are assumed to be half as contagious as natural varicella cases, i.e. $\alpha=0.5$ [14].

### Individuals infected with varicella (I or I^*^) recover at a constant rate $\boldsymbol{\gamma}$, where $\boldsymbol{1/}\boldsymbol{\gamma}$ is assumed to be equal to 3 weeks and represents the generation time associated to VZV infection. Once recovered from varicella, both unvaccinated individuals who experienced natural varicella and vaccinated individuals who experienced breakthrough varicella gain lifelong immunity to VZV re-infection and become susceptible to HZ (ZS_1_ and ZS_1_^*^, respectively).

### Individuals who become susceptible to HZ after natural varicella (ZS_i_) are assumed to develop HZ disease at a rate $\boldsymbol{\rho}$, which is assumed to depend on individuals’ chronological age$\boldsymbol{a}$, the number of VZV exposure episodes they have experienced *i*, and the time elapsed since their last VZV exposure $\boldsymbol{\tau}$. Specifically, the VZV reactivation rate is modelled as follows:

### $\boldsymbol{\rho}_{\boldsymbol{i}}\boldsymbol{(a,\tau)=}\boldsymbol{\rho}_{\boldsymbol{0}}\boldsymbol{q}^{\boldsymbol{(i-1)}^{\boldsymbol{2}}}\boldsymbol{e}^{{\boldsymbol{\theta}_{\boldsymbol{a}}\boldsymbol{(a-}\boldsymbol{a}_{\boldsymbol{0}}\boldsymbol{)}}^{\boldsymbol{+}}}\boldsymbol{e}^{\boldsymbol{\theta}_{\boldsymbol{\tau}}\boldsymbol{\tau}}$ (2)

### where $\boldsymbol{\rho}_{\mathbf{0}}$ represents the risk of developing HZ for individuals who have just recovered from varicella infection ($\boldsymbol{i}$=1, $\boldsymbol{\tau}$=0 and $\boldsymbol{a<}\boldsymbol{a}_{\mathbf{0}}$=45 years), $\boldsymbol{q}$ is the parameter shaping the reduction of the HZ risk due to re-exposures to VZV, $\boldsymbol{\theta}_{\boldsymbol{a}}$ is the parameter shaping the increase of HZ risk with chronological age *a* and $\boldsymbol{\theta}_{\boldsymbol{\tau}}$ is the parameter shaping the increase of HZ risk with time since last exposure to VZV, namely $\boldsymbol{\tau}$.

Individuals who become susceptible to HZ after experiencing breakthrough varicella (ZS_i_^*^) are assumed to develop HZ at a rate$\chi\rho_{i}(a,\tau)$, where $\chi$ denotes the reduction of HZ risk in vaccinated individuals. Individuals successfully immunized against varicella (VP_i_) are assumed to develop HZ from the varicella vaccine strain at a rate$\chi\rho_{i}(a,\tau)$, as well. In this case $\chi\rho_{0}$ represents the risk of developing HZ for individuals who have just vaccinated against varicella ($i$=1, $\tau$=0 and $a<a_{0}$=45 years).

### Re-exposures to VZV of HZ susceptible individuals (ZS_i_, ZS_i_^*^, VP_i_) are called boosting events and occur at a rate$\boldsymbol{z}\boldsymbol{\lambda}\left( \boldsymbol{a,t} \right)$, that is assumed proportional to the FOI $\boldsymbol{\lambda}\left( \boldsymbol{a,t} \right)$ through a coefficient $\boldsymbol{z}$∈ [0,1]. When an individual experiences a boosting event, the value of *i* associated with this specific individual is incremented by one.

### HZ vaccination is assumed to occur at a constant coverage *c_HZ_*. In this case we assume that a fraction *p_HZ_* of vaccinated individuals develop immunity against VZV reactivation into HZ, where *p_HZ_* represents the HZ vaccine efficacy.

After developing HZ or being successfully immunized against HZ, individuals are assumed to gain life-long immunity to VZV reactivation (ZR).

### In the proposed individual-based model, information on individuals’ age $\boldsymbol{a}$ and their epidemiological status (which include the number of VZV exposure episodes *i*, and time elapsed since last VZV exposure $\boldsymbol{\tau}$) is tracked and updated over time separately for each individual represented in the simulated synthetic population.

### Time-varying average contact matrices $\boldsymbol{C}_{\boldsymbol{a}\boldsymbol{a}^{\boldsymbol{'}}}\left( \boldsymbol{t} \right)$were derived from age specific contact patterns estimated for Italy [4], by applying the same approach used in previous works [13,15]. The free parameters in model PI are the following ones: $\boldsymbol{\beta,z,\rho}_{\mathbf{0}}\boldsymbol{,} \boldsymbol{q, \theta}_{\boldsymbol{a}}$,$\boldsymbol{\theta}_{\boldsymbol{\tau}}$. More details on this model can be found in [8,9,13].

### Model TI

In model TI, demographic transitions, VZV transmission, vaccination against varicella and HZ are modelled as in model PI (see Fig S3), while VZV reactivation into HZ is modelled in a different way. Specifically, after recovery from varicella infection (either natural or breakthrough, I and I^*^ in Figure S3), individuals acquire a temporary but full immunity against VZV reactivation (Z_p_ or Z_p_^*^). However, after $1\boldsymbol{/}\delta$ years on average, the acquired immunity wanes and individuals become susceptible to HZ (ZS or ZS^*^). The risk $\rho$ of VZV reactivation for individuals who become susceptible to HZ after the recovery from natural varicella infection (ZS) depends only on their chronological age $a$ and it is modelled as follows:

$\rho\left( a \right)=\omega e^{-\varphi a}+a^{\eta}\pi$ (3)

The first term defining the VZV reactivation rate in Equation (3) decreases with age, while the second term increases with age. This formulation is sufficiently flexible to reproduce, in principle, a higher VZV reactivation rate in young children due to due incomplete development of immunocompetence and the increase in VZV reactivation rate with age as consequence of immunosenescence occurring in the elderly [11,16,17].


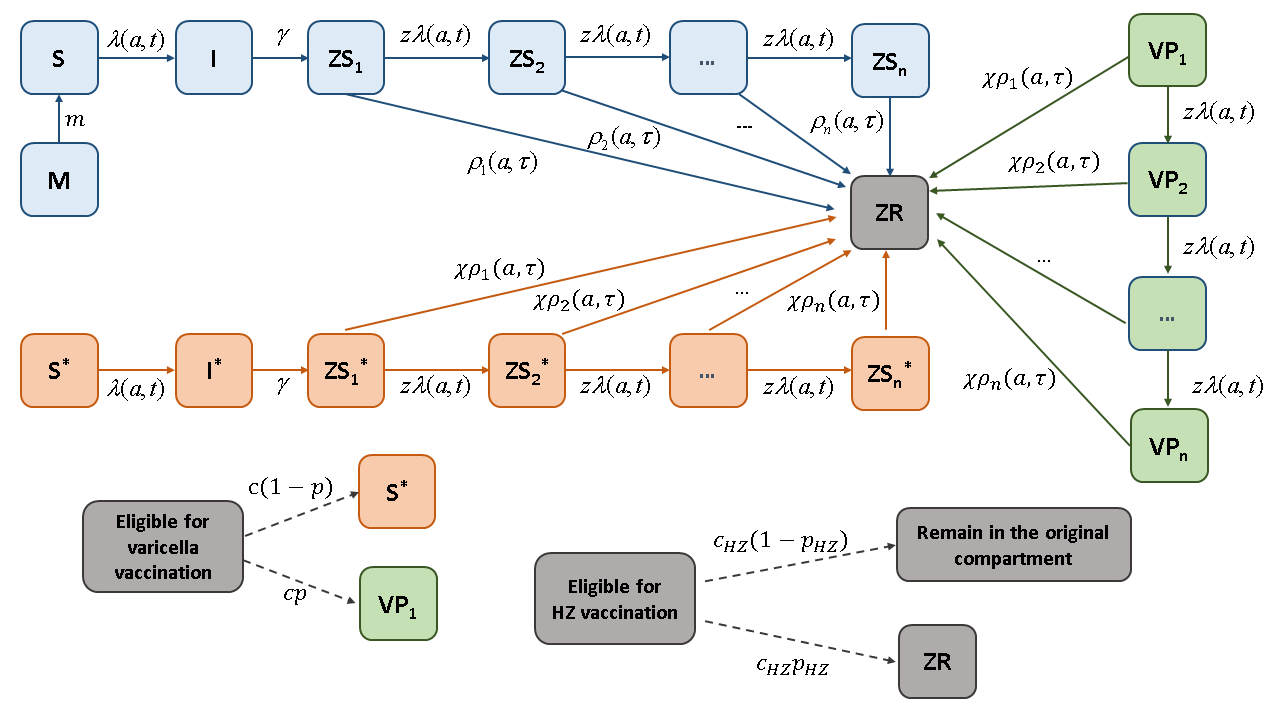

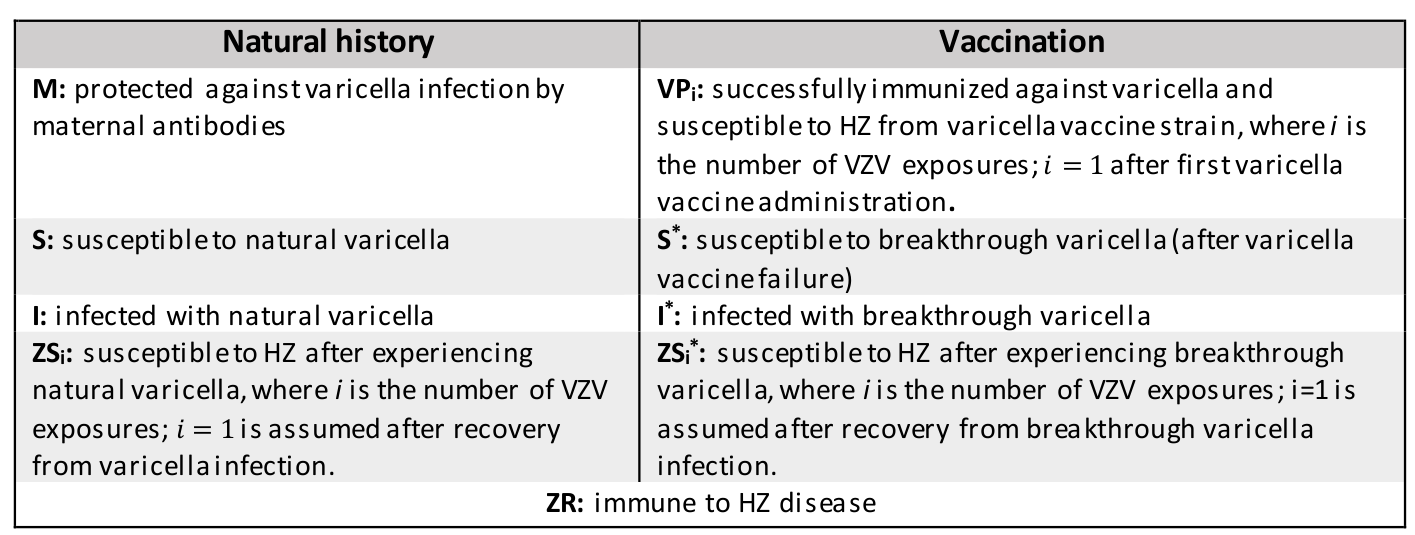


**Figure S2: Epidemiological structure of model PI.** Acronyms used for different epidemiological status are summarized in the legend above. Different colors are used to indicate independent paths that can bring to develop HZ disease: VZV reactivation after infection with natural varicella (light blue), VZV reactivation after infection with breakthrough varicella (orange), VZV reactivation from varicella vaccine strain (green). Solid arrows indicate rates; dotted arrows represent probabilities adopted to simulate different outcomes of vaccination of individuals.

Legend

Analogously to model PI, individuals who are susceptible to HZ after experiencing breakthrough varicella (ZS^*^) and those who are successfully immunized against varicella (VP), are assumed to reactivate VZV into HZ at a rate $\chi\rho\left( a \right)$, where $\chi$ denotes the reduction of HZ risk in vaccinated individuals.

### Re-exposures of HZ susceptible individuals (ZS, ZS^*^, VP) to VZV, occurring at a rate$\boldsymbol{z}\boldsymbol{\lambda}\left( \boldsymbol{a,t} \right)$, are assumed to confer a temporary full protection against HZ, lasting on average $\boldsymbol{1/\delta}$ years. In the individual-based model, information on individuals’ age $\boldsymbol{a}$ and their epidemiological status is tracked and updated over time separately for each individual represented in the simulated synthetic population. The free parameters of model TI are the following ones: $\boldsymbol{\beta,z, \omega,\varphi,\eta,\pi}$ and $\boldsymbol{\delta}$.


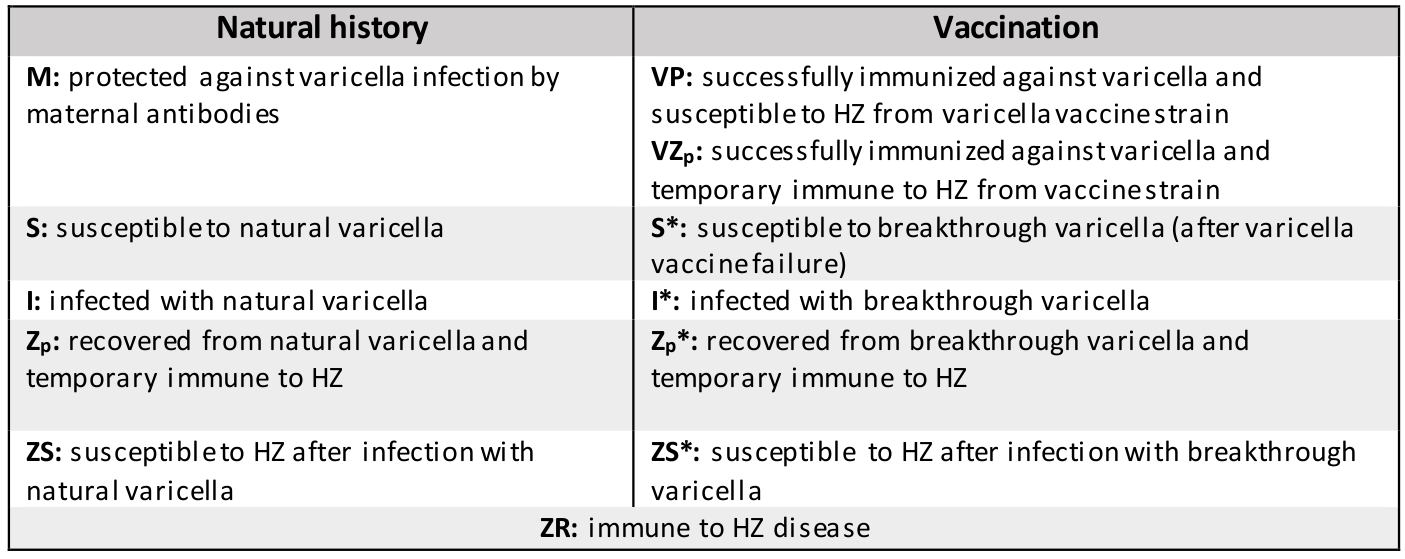

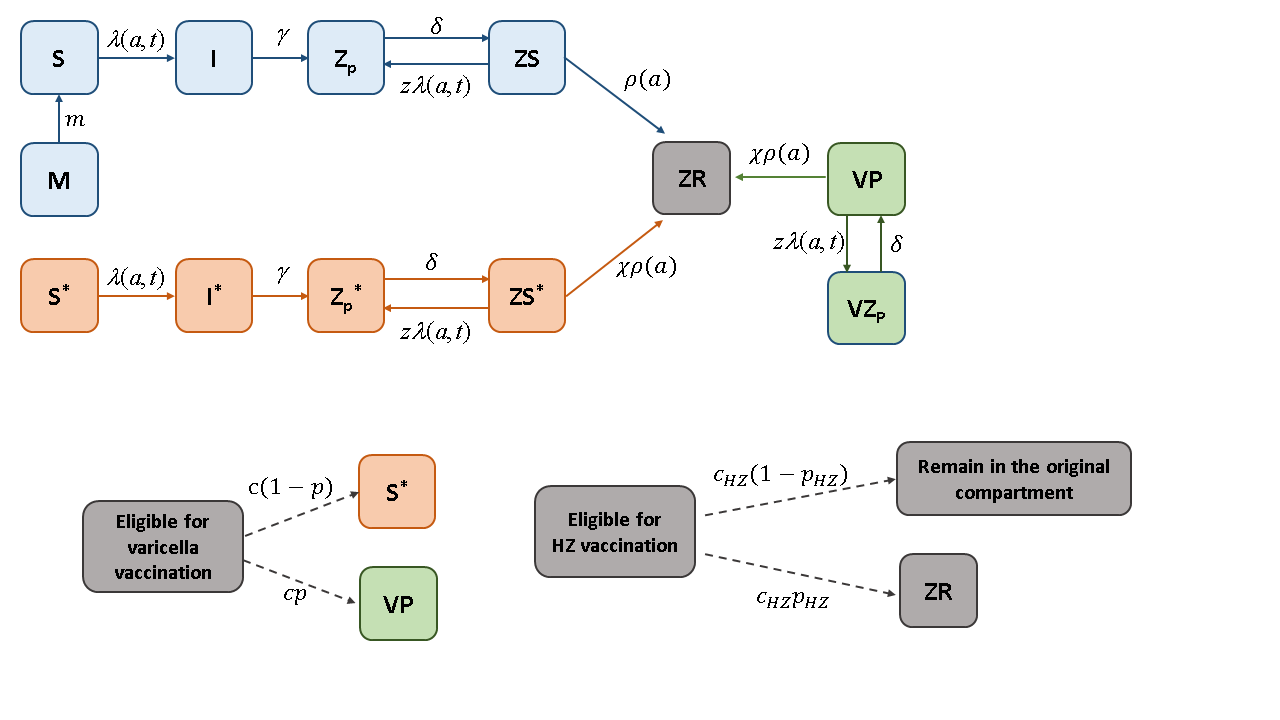


**Figure S3: Epidemiological structure of model TI.** Acronyms used for different epidemiological status are summarized in the legend above. Different colors are used to indicate independent paths that can bring to develop HZ disease: VZV reactivation after infection with natural varicella (light blue), VZV reactivation after infection with breakthrough varicella (orange), VZV reactivation from varicella vaccine strain (green). Solid arrows indicate rates; dotted arrows represent probabilities adopted to simulate different outcomes of vaccination of individuals.

Legend

### Running these models is computationally intensive. In particular, model PI requires tracking HZ susceptibility states by age, time since last exposure to VZV, and cumulative number of re-exposures experienced by individuals. This means that by considering 100 one-year age groups, a mean number of lifetime re-exposures of about 7 (as estimated by previous works [13]) and a yearly discretization for the time since last exposure, the number of possible states for individuals susceptible to HZ amounts to at least 7×100×100/2 = 35,000 (division by 2 accounts for the time since last exposure being always smaller than chronological age). This number possibly underestimates the required number of equations in the considered model (indeed, some individuals may experience more than 7 re-exposure episodes). These simple calculations suggest that the implementation of a PI model with a deterministic ordinary differential equations (ODE) system would lead to run a model with more than 35,000 different equations for about 200 years, which would be too computationally intensive, especially when adopting an MCMC approach to estimate the free model parameters. For these reasons, a stochastic individual-based modelling approach was chosen for both model PI and TI, which resulted to be, as a matter of fact, much less computationally intensive than simulating the model with an ODE deterministic approach.

### Model calibration

Models’ calibration was performed using a Markov Chain Monte Carlo (MCMC) approach applied to the likelihood of observing the VZV seroprevalence profile reported for 1996-1997 [5], and the age-specific HZ incidence reported for 2004 [6]. The two models were calibrated separately following the same procedure briefly described hereafter.

At each iteration, the likelihood of a new candidate vector of parameters Θ is evaluated and the parameter set is accepted or not based on the standard Metropolis-Hastings algorithm [18]. At each iteration, the last accepted parameter set is repeated in the MCMC sample. For each candidate parameter set, the population of the model is initialized according to the age structure observed in 1900 and the individuals’ epidemiological status is assigned according to the fraction associated with the equilibrium solution corresponding to the considered parameter set (in the absence of vaccination). In particular, for each parameter set, the equilibrium solution is obtained by running the demographic and transmission model with constant fertility and mortality rates fixed to those recorded in 1900, and by initializing the system with ten individuals infected with VZV in a fully susceptible population. From 1900 on, the dynamics of VZV is simulated by running the model with time-varying crude birth, migration, and age-specific mortality rates. The likelihood associated to the parameter set is computed by comparing the simulated and observed varicella seroprevalence in 1996 and HZ incidence in 2005. Candidate parameter sets are accepted or not following the standard Metropolis-Hastings algorithm, based on 22,000 iterations and a burn-in period of 2,000 iterations [18].

The likelihood function used in the MCMC calibration procedure is defined as the product between the binomial likelihood $\mathcal{L}_{VZV}$ of the observed varicella serological profile by age in 1996 [5] and the Poisson likelihood $\mathcal{L}_{HZ}$ of the observed HZ incidence profile by age in 2005-2006 [6].

Specifically, we assume that

$$\mathcal{L}_{VZV}\left( n_{m}, r_{m} \right|\Theta)=\prod_{m} \frac{n_{m}!}{r_{m}!\left( n_{m}-r_{m} \right)!}\left( p_{m}\left( \Theta\right) \right)^{r_{m}}{(1-p_{m}(\Theta))}^{{n_{m}-r}_{m}}$$

and that

$$\mathcal{L}_{HZ}\left( k_{j} \right|\Theta)=\prod_{j\in J} \frac{e^{-\eta_{j}\left( \Theta\right)}{{(\eta}_{j}(\Theta))}^{k_{j}}}{k_{j}!}$$

where:

- $\Theta=\left\{ \begin{matrix} \left( \beta,z,\rho_{0}, {q, \theta}_{a}\mathbf{,} \theta_{\tau} \right) \text{for model PI} \\ \left( \beta,z,\omega,\varphi,\eta,\pi,\delta\right) \text{for model TI} \end{matrix} \right.$ ;
- *m* runs over age classes considered in the serological profile [5];
- $n_{m}$is the number of individuals tested in the age class *m* [5];
- $r_{m}$is the number of seropositive individuals found within the age class *m* [5];
- $p_{m}(\Theta)$ is the fraction of VZV seroprevalent individuals in the age class *m*, as simulated by the model (PI or TI) in 1996 with parameter set $\Theta$;
- *J* is the set of age groups in the HZ incidence profile [6];
- $k_{j}$is the total number of HZ cases in the *j*-th age group observed in [6];
- $\eta_{j}\left( \Theta\right)$ is the number of HZ cases in the age group *j*, simulated by model (PI or TI) in 2005 in a population of the same size as that analyzed in [6], with the parameter set $\Theta$.

Free parameters include one parameter shaping the force of infection of varicella ($\beta$), one determining the fraction of contacts with VZV-infectious individuals that result in effective boosting ($z$), and a set of parameters shaping the VZV reactivation rate: ($\rho_{0}, {q, \theta}_{a}$**,**$\theta_{\tau}$) for model PI, and $(\omega,\varphi,\eta,\pi$) for model TI. In Model TI, an additional free parameter is represented by the duration of the complete protection conferred by a boosting event ($\delta$).

In both models, the relative risk of VZV reactivation among vaccinated individuals is assumed to be 85% lower than the one of unvaccinated individuals ($\chi=0.15$) [19]. A sensitivity analysis on the assumed value for $\chi$ is conducted in section 8.2.2.

Routine varicella vaccination is given with a 2-dose schedule (15 months, 5-6 years). HZ vaccine is offered to the elderly (65 years), either alone or in combination with an initial catch-up campaign (66-75 years). Varicella vaccine efficacy per dose is set to 80%, which implies an efficacy of 96% after two doses, while it is set to 50% for one dose of HZ vaccine.

Estimates obtained for free model parameters of model PI and TI, are summarized in Tables S1 and S2 respectively, and shown along with epidemiological parameters that were fixed on the basis of previous epidemiological and modeling studies.

Both model structures provided a good fit to the VZV serological profile observed in Italy in 1996-1997, including the drop in the percentage of seropositive after the first year of age, at the end of the maternal antibody protection (Figure S4, panels on the left). Also for HZ, both models are capable to reproduce the HZ incidence by age observed in 2004 (Figures S4, panels on the right), even though with some small differences.


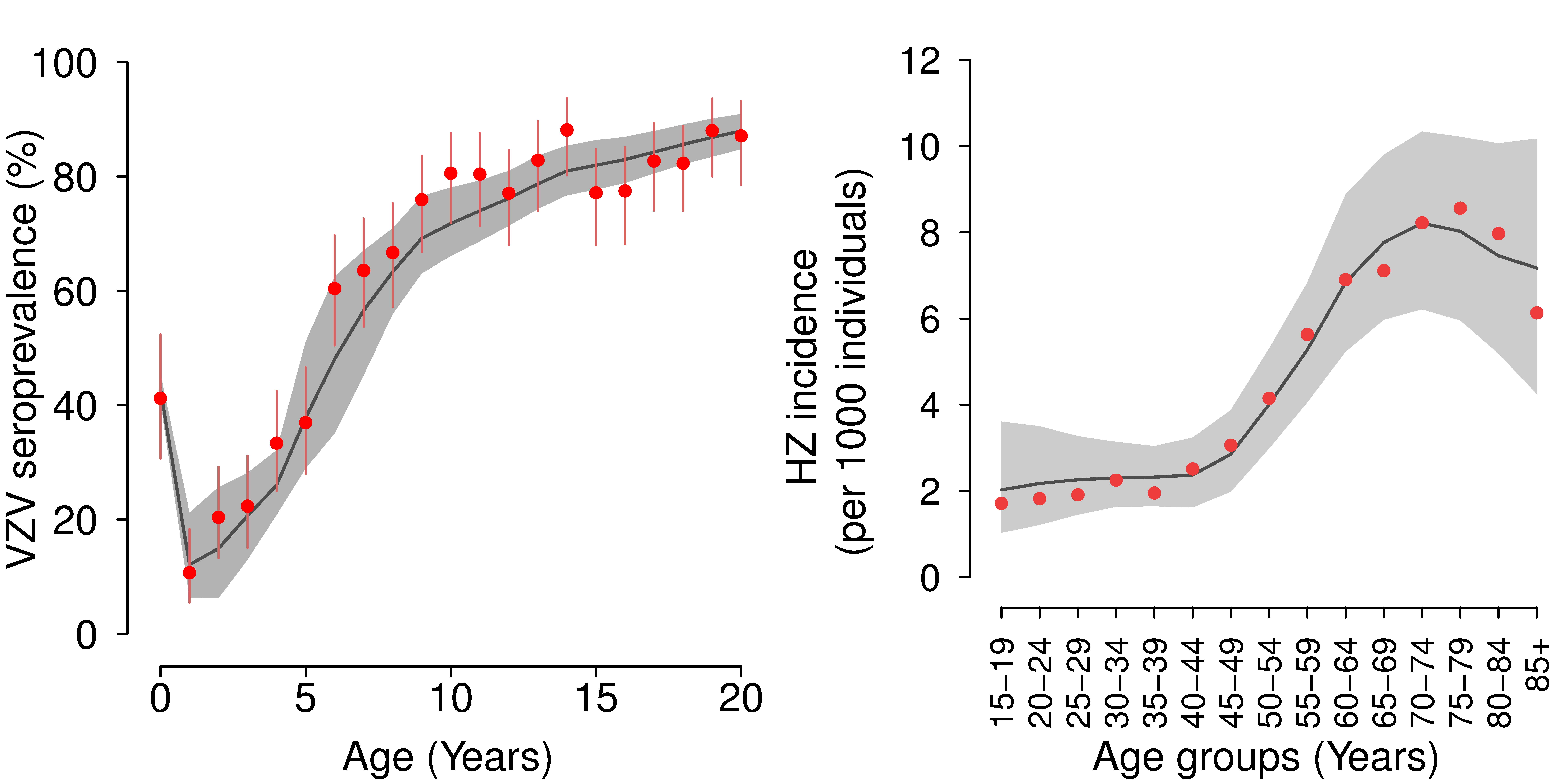


**Figure S4: Model fit to available epidemiological data.** On the top row, Model TI fit to varicella seroprevalence (on the left) and to HZ incidence (on the right). On the bottom row, Model PI fit to varicella seroprevalence (on the left) and to HZ incidence (on the right).

**Table S1: Epidemiological parameters: model PI**

| Parameters | Description | Value (mean [95%CI]) | Unit of measurement | Assumed/  Estimated |
| --- | --- | --- | --- | --- |
| $\boldsymbol{1/\gamma}$ | Generation time | 3 | weeks | Assumed [8,10,17,20] |
| $\boldsymbol{\beta}$ | VZV transmission rate | 1.01∙10^5^ [0.91∙10^5^-1.11∙10^5^] | weeks^-1^ | Estimated |
| $\boldsymbol{z}$ | Boosting efficacy | 0.6 [0.37-0.92] |  | Estimated |
| $\boldsymbol{\rho}$ | VZV reactivation rate | Defined as:  $\rho_{i}(a,\tau)=\rho_{0}q^{{(i-1)}^{2}}e^{{\theta_{a}(a-a_{0})}^{+}}e^{\theta_{\tau}\tau}$ | years^-1^ | Estimated |
|  |  | $\rho_{0}$: 3.5∙10^-3^[1.3∙10^-3^-8.1∙10^-3^] |  |  |
|  |  | $q$: 0.25 [0.04-0.48] |  |  |
|  |  | $\theta_{a}$: 6.5∙10^-2^ [2.2∙10^-2^-10.2∙10^-2^] |  |  |
|  |  | $\theta_{\tau}$: 3∙10^-2^[0.2∙10^-2^-5.7∙10^-2^] |  |  |
| $\boldsymbol{\chi}$ | Scale factor for the VZV reactivation rate of vaccinated individuals | Base case: 0.15  PSA: 0.08 and 0.23 |  | Assumed  [19] |
| *c* | Varicella vaccination coverage | Base case: 80%  PSA: 75% and 95% |  | Assumed |
| *p* | Varicella vaccine efficacy (per dose) | 80% |  | Assumed  [21] |
| *c_HZ_* | HZ vaccination coverage | 60% |  | Assumed |
| *p_HZ_* | HZ vaccine efficacy | 50% |  | Assumed [22] |

**Table S2: Epidemiological parameters: model TI**

| Parameters | Description | Value (mean [95%CI]) | Unit of measurement | Assumed/  Estimated |
| --- | --- | --- | --- | --- |
| 1/$\boldsymbol{\gamma}$ | Generation time | 3 | weeks | Assumed [8,10,17,20] |
| $\boldsymbol{\beta}$ | VZV transmission rate | 1.04∙10^5^[0.91∙10^5^-1.15∙10^5^] | weeks^-1^ | Estimated |
| $\boldsymbol{z}$ | Boosting efficacy | 0.62 [0.06,0.98] |  | Estimated |
| $\boldsymbol{1/\delta}$ | Average duration of immunity to zoster after varicella infection | 113.5  [80.5,159] | years | Estimated |
| $\boldsymbol{\rho}$ | VZV reactivation rate | Defined as:  $\rho\left( a \right)=\omega e^{-\varphi a}+a^{\eta}\pi$ | years^-1^ | Estimated |
|  |  | $\omega$: 0.26 [0.05-0.56] |  |  |
|  |  | $\varphi$: 0.10[0.05,0.14] |  |  |
|  |  | $\eta$: 2.35 [2,2.68] |  |  |
|  |  | $\pi$: 0.33 [0.08,0.93] |  |  |
| $\boldsymbol{\chi}$ | Scale factor for the VZV reactivation rate of vaccinated individuals | Base case: 0.15  PSA: 0.08 and 0.23 |  | Assumed  [19] |
| *c* | Varicella vaccination coverage | Base case: 80%  PSA: 75% and 95% |  | Assumed |
| *p* | Varicella vaccine efficacy (per dose) | 80% |  | Assumed  [21] |
| *c_HZ_* | HZ vaccination coverage | 60% |  | Assumed |
| *p_HZ_* | HZ vaccine efficacy | 50% |  | Assumed [22] |

### Model’s estimates and projections

The algorithm described above, based on MCMC and standard Metropolis-Hastings [18], provides 20,000 MCMC samples from the posterior distribution of the free model parameters. These distributions were used to 1) estimate the posterior means and 95% credible intervals (CI) of the free parameters, 2) randomly sample 1,000 parameters configurations that were used to investigate the VZV infection dynamics in the population, and 3) simulate the different vaccination strategies considered. Distributions of model outcomes are described in terms of average outcomes and 95% of prediction intervals (95% PI).

In order to test whether in the proposed analysis the parameters space has been sufficiently explored, we tested, for each considered vaccination strategy, how the distribution of the net monetary benefits (NMB) changed by including an increasing number of model realizations from 1 to 1000. For this analysis, a time horizon of 85 years and a discounting rate of 3% were considered.

In particular, for each of the five considered vaccination programs (V_R_, HZ_R_, HZ_R+CU_,V_R_HZ_R_, V_R_HZ_R+CU_), we compared the 2.5%, 25%, 50%, 75%, and 97.5% quantiles of the distribution of NMB, as obtained by using 1, 2, 3, …, 1000 realizations randomly chosen from 1000 model outcomes. The experiment was repeated 100 times to allow inclusion of different samples across different experiments when a number of realizations <1000 was considered.

The results obtained for model PI are shown in the Figure S5. Similar results were obtained for model TI (not shown).


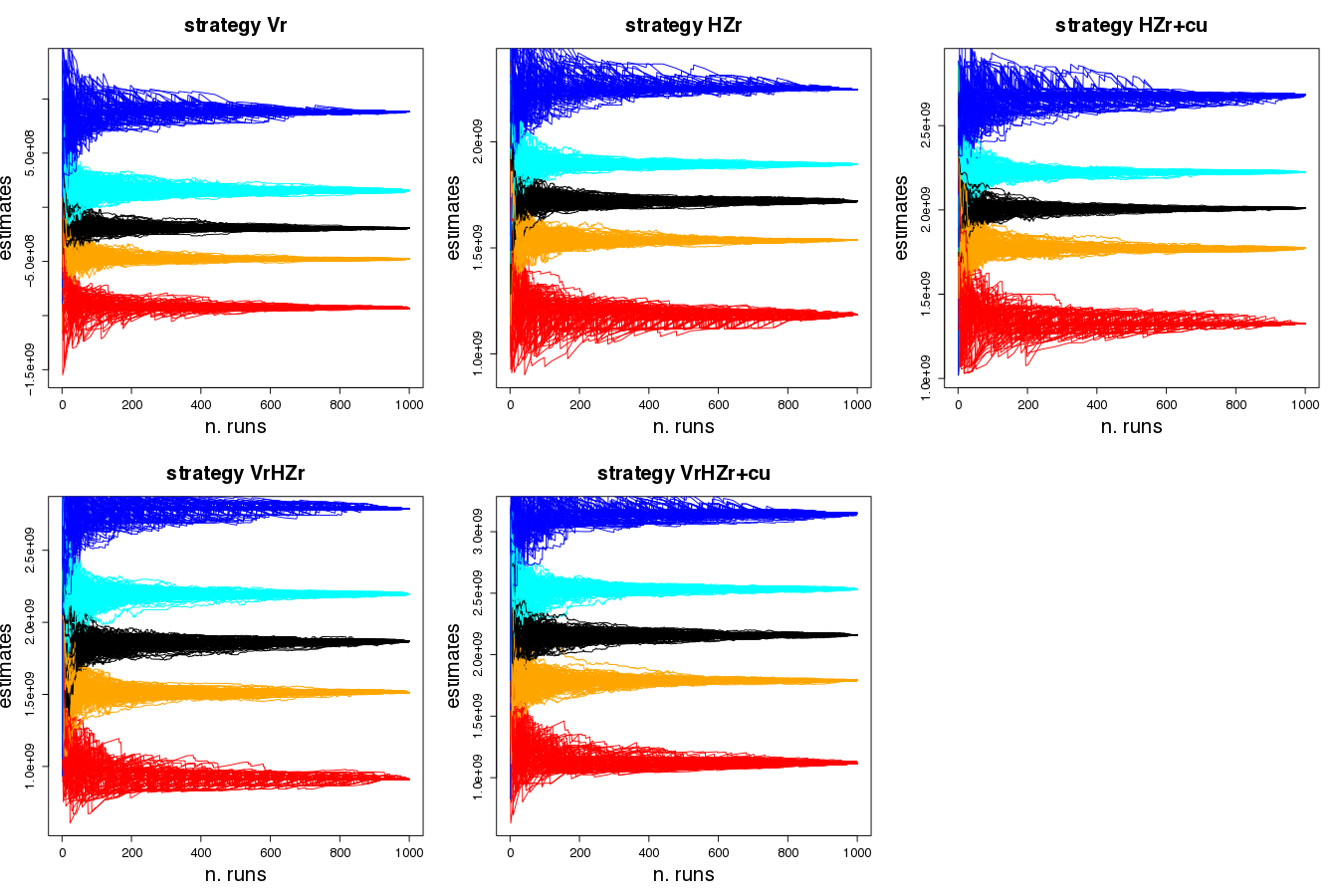


**Figure S5: NMB and the number of model realizations.** Distribution of NMB associated with model PI and different vaccination strategies: 2.5%, 25%, 50%, 75%, and 97.5% quantiles are respectively shown in red, orange, black, cyan, blue; different lines with the same color refers to different repetitions of the experiment.

Our results suggest that the distribution of NMB is rather stable when considering more than 600 realizations. Boxplots of the distribution of NMB obtained by considering one single experiment, using the first 100, 200, and 1000 simulations are shown in Figure S6.

###

**Figure S6: NMB and the number of model realizations.** Box plots of the distribution of NMB (2.5%, 25%, 50%, 75%, 97.5% quantiles) of different vaccination strategies as resulting when considering the first 100, 200, and 1000 model realizations obtained with model PI.

### Past VZV epidemiology in Italy (1900-2015)

During the last century, the Italian population underwent a fertility transition comparable to that observed for other European countries. According to both models, the overall decreasing trend of the birth rate observed in Italy since 1900 has reduced the proportion of children in the overall population leading to a reduction of VZV circulation. In particular, according to model estimates during the considered period, the average age at varicella infection has increased and the fraction of VZV seropositive individuals has decreased at all ages (Figure S7 and S8). The lower VZV circulation over time led to an overall reduction of the frequency of boosting events, whose impact on HZ incidence is different depending on the formulation used to model such boosting. According to model PI, the reduction of boosting results in an increase of 143% (95% PI: 72%-206%) in the total HZ incidence between 1900 to 2015. Instead, the increase predicted by model TI is significantly lower, namely, 55% (95% PI: 24%-90%). On the other hand, the increase in the average age at VZV reactivation, which is mainly ascribable to population ageing, has the same magnitude under both models. These results suggest that, under model PI, the dynamics of HZ are much more sensitive to changes in varicella circulation than under model TI.

**Figure S7:** **Change in VZV epidemiology in Italy (1900-2015), according to model TI.** From top left to bottom left, clockwise, we report the age-specific seroprevalence (in percentage) for different decades, the average age at varicella over time, the average age at VZV reactivation over time, and the age-specific HZ incidence (per 1,000 individuals) for different decades.

**
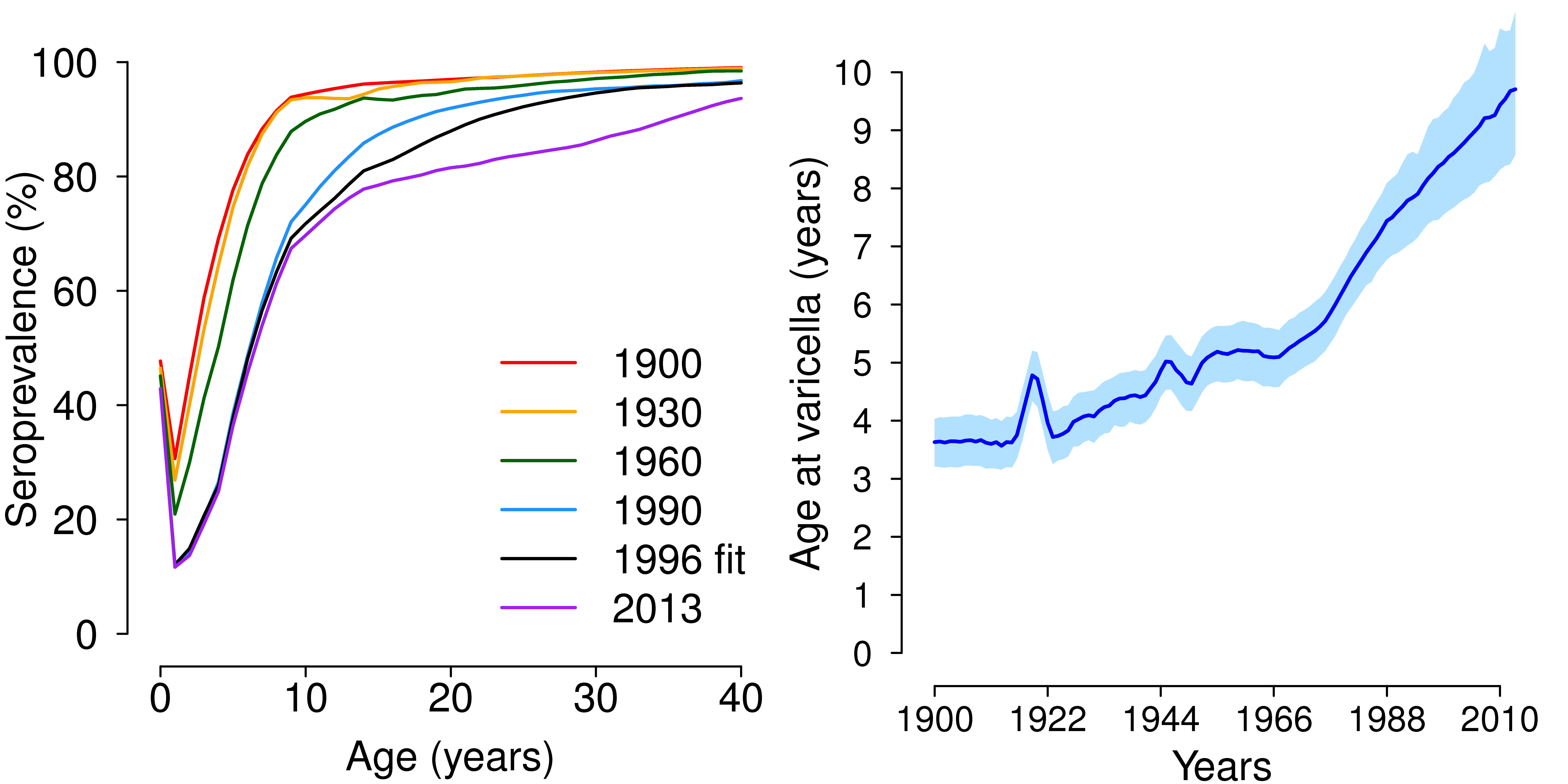
**

**
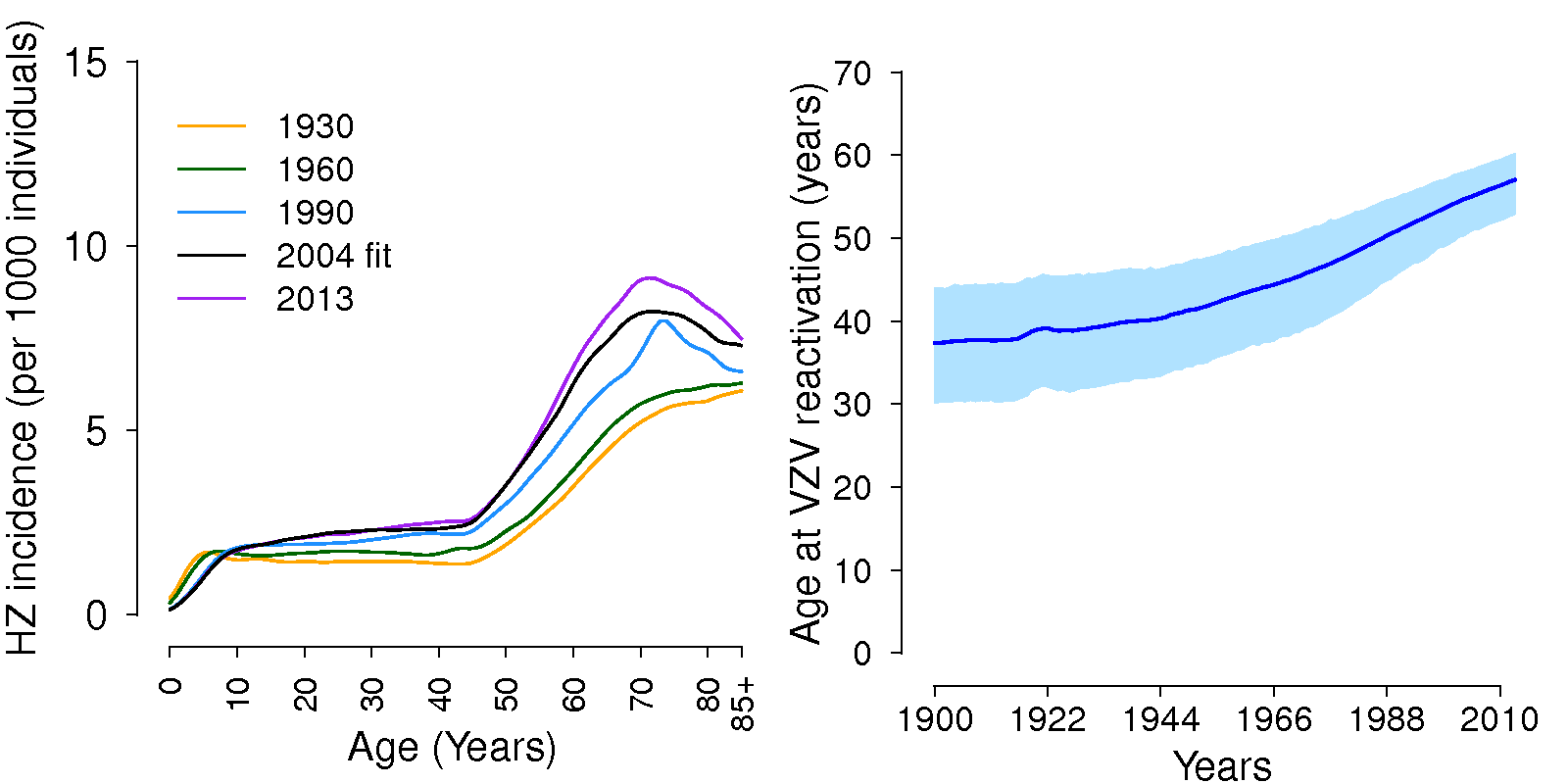
**

**Figure S8:** **Change in VZV epidemiology in Italy (1900-2015), according to model PI.** From top left to bottom left, clockwise, we report the age-specific seroprevalence (in percentage) for different decades, the average age at varicella over time, the average age at VZV reactivation over time, and the age-specific HZ incidence (per 1,000 individuals) for different decades.

### Future VZV epidemiology in Italy (2015-2100)

### Model predictions on varicella and HZ under a routine varicella program (V_R_) and a combined program (V_R_HZ_R+CU_) are shown in Figures S9 and S10 considering both models’ structures.


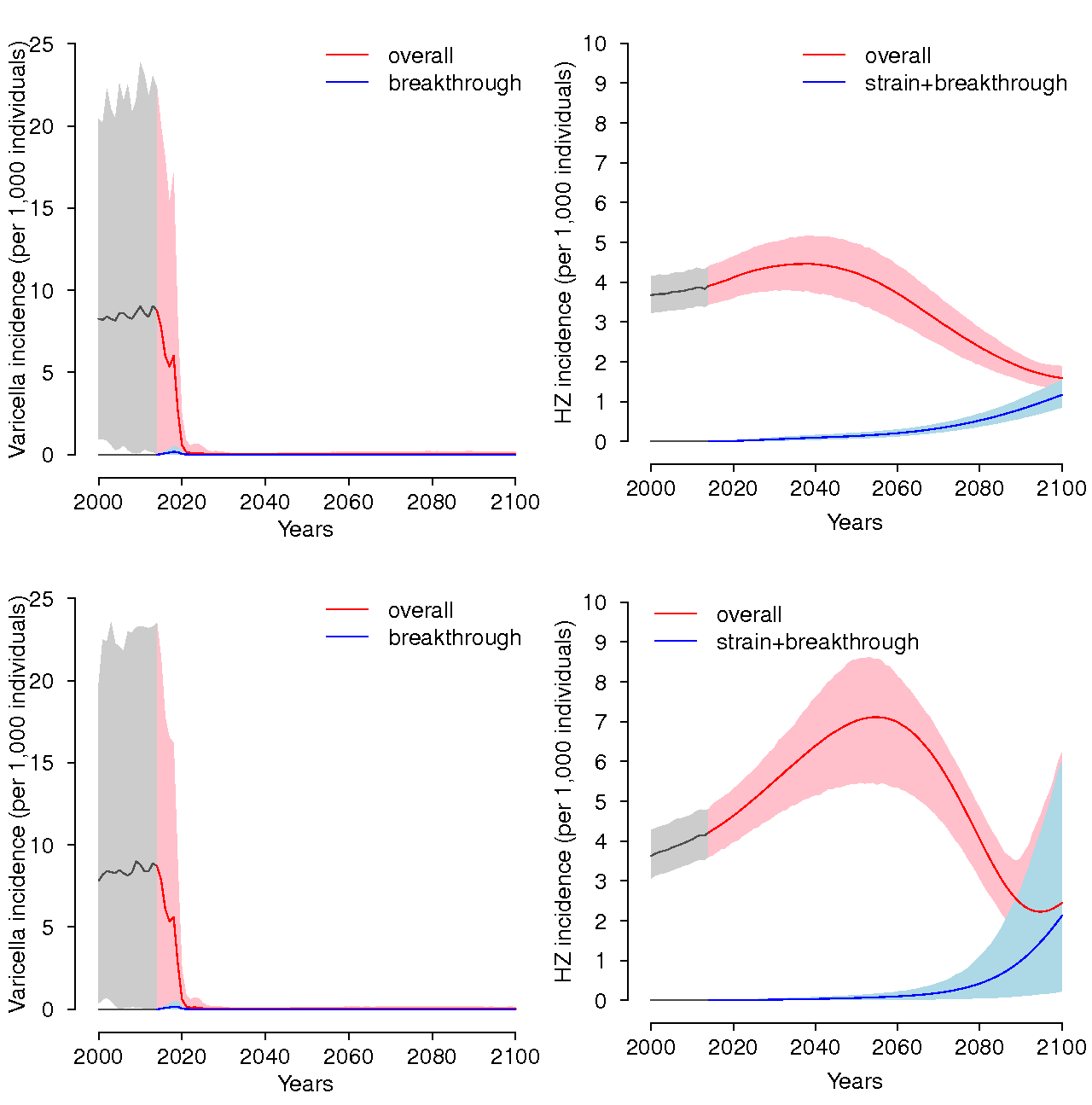


**Figure S9:** Model predictions on varicella (left) and HZ (right) incidence under V_R_ vaccination. TI model top row, PI model bottom row. Routine varicella vaccination is given with a 2-dose schedule (15 months, 5-6 years), with efficacy p=80% and coverage c=80%; HZ risk reduction for vaccines is assumed to be $\chi$=0.15; HZ vaccine is administered at 65 years of age (66-75y catch-up), with efficacy *p_HZ_* =50% and coverage *c_HZ_* =60%.

**
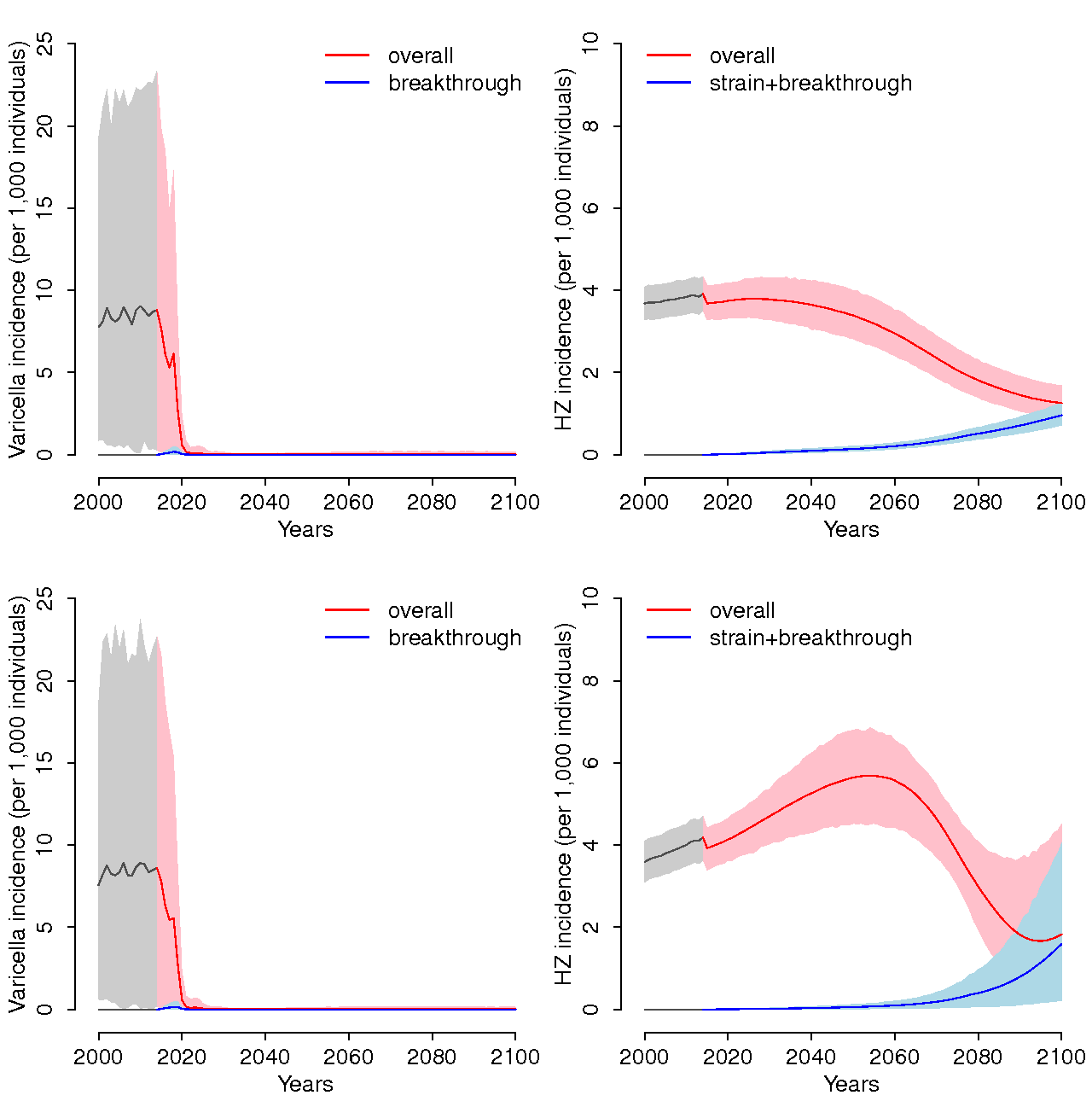
**

**Figure S10:** Model predictions on varicella (left) and HZ (right) incidence under scenario V_R_HZ_R+CU_ (i.e., varicella and HZ vaccination). TI model top row, PI model bottom row. Routine varicella vaccination is given with a 2-dose schedule (15 months, 5-6 years), with efficacy p=80% and coverage c=80%; HZ risk reduction for vaccines is assumed to be $\chi$=0.15; HZ vaccine is administered at 65 years of age (66-75y catch-up), with efficacy *p_HZ_* =50% and coverage *c_HZ_* =60%.

### Cost-effectiveness analysis

### *Decision tree for varicella infection*

All individuals at birth are protected against VZV infection by passive transfer of maternal antibodies. Maternal antibodies protection last 6 months on average, after which individuals become susceptible to varicella infection. In case there isn’t any varicella vaccination program, individuals may get infected with VZV and acquire natural varicella (NV). Individuals with NV see a general practitioner (GP) according to the number of GP consultations per NV case (at the cost of a GP consultation per NV, different for children up to 15 years old and for the others) and get treatment for the infection (at the cost of a GP treatment per NV, different for children up to 15 years old and for the others) (Figure S11). After the treatment, individuals may recover or get worse and be hospitalized, according to the age-specific NV hospitalization rate. The cost of hospitalization is specific for NV and varies by age. Hospitalized NV cases may in turn recover or die, according to the age-specific NV case fatality rate.

In case there is an on-going varicella vaccination program, individuals who are susceptible to VZV and eligible for vaccination get vaccinated with two doses at the cost of varicella vaccination (plus the administration costs). Notwithstanding the vaccination, these individuals may still acquire breakthrough varicella (BV). Individuals with BV see a doctor according to the number of GP consultation per BV case (at the same cost of a GP consultation per NV) and get treatment for the infection (at the same cost of a GP treatment per NV). Again, after the treatment, individuals may recover or get worse and be hospitalized, according to the age-specific BV hospitalization rate (which is proportionally lower than the hospitalization rate for NV). The cost of hospitalization for BV is assumed equal to the one for NV and varies by age. Hospitalized BV cases may in turn recover or die, according to the age-specific BV case fatality rate (which is proportionally lower than the case fatality rate for NV).

**Figure S11:** **Decision tree for natural and breakthrough varicella infection.**

### *Decision tree for HZ infection*

All individuals who get infected with VZV successively become susceptible to HZ infection (also individuals vaccinated for varicella may become susceptible to HZ). In case there is no HZ vaccination program, individuals may acquire HZ, a proportion of which may develop post-herpetic neuralgia (PHN) (Figure S12).

Individuals with HZ (and no PHN) see a GP and get treatment for the infection (at the outpatient cost for HZ), after which they may recover or get worse and be hospitalized, according to the age-specific HZ hospitalization rate. The cost of hospitalization is specific for HZ and varies by age. Hospitalized HZ cases may in turn recover or die, according to the age-specific HZ case fatality rate.

Individuals with PHN see a GP and get treatment for the infection (at the outpatient cost for PHN), after which they may recover or get worse and be hospitalized, according to the age-specific PHN hospitalization rate. The cost of hospitalization is specific for PHN and varies by age. Hospitalized PHN cases may in turn recover or die, according again to the age-specific HZ case fatality rate.

Finally, if there is an on-going HZ vaccination program, individuals who are susceptible to HZ and eligible for vaccination get vaccinated with one dose at the cost of HZ vaccination (plus the administration costs). We assume that individuals vaccinated against HZ do not acquire HZ.

**Figure S12:** **Decision tree for HZ infection and PHN.**

### *Measures of effectiveness*

QALY lost by age for case of infection and for death were derived in a similar way to what presented in van Hoek et al. (2012) [23]. Quality of Life weights for varicella were derived from Brisson and Edmunds (2003) [24]. QALY lost by age for NV were calculated based on an average duration of the infection of seven days, with a distinction between severe NV cases (more than 50 spots) and mild NV cases (less than 50 spots) [25]. The QALY loss for mild cases was scaled by a proportionality factor assumed equal to 25% [21,26]. QALY lost by age for BV were instead calculated for an average duration of the infection of six days, with a distinction between severe BV cases (more than 50 spots) and mild BV cases (less than 50 spots) [25]. Again, QALY loss for mild cases was scaled by a proportionality factor of 25%.

QALY lost by age for HZ and PHN were taken from van Hoek et al. (2012) [23].

QALY loss for death per infection was obtained by multiplying the general QALY loss for death for the mortality rate per infection. Since our model assumed that infection-related deaths were possible only for the hospitalized cases, the mortality rate per infection was calculated by multiplying the hospitalization rate per infection by the case fatality rate per infection.

**Table S3: Impact on the burden of illness, with 3% discount rate.** Cumulative number of infection cases and disease-related deaths, reported separately for varicella (natural and breakthrough) and HZ (with and without PHN), by vaccination policy, model and time horizon (TH=25, 50, 85). All outcomes are reported with a 3% discount rate for both benefits and costs.

| **Model TI** | | | | | **Model PI** | | | | |
| --- | --- | --- | --- | --- | --- | --- | --- | --- | --- |
| Policy | VAR cases | HZ cases | VAR deaths | HZ deaths |  | VAR cases | HZ cases | VAR deaths | HZ deaths |
| **TH=25** | | | | | | | | | |
| No Vacc. | 8 682 999 | 4 359 928 | 143 | 2 540 | No Vacc. | 8 652 633 | 5 191 376 | 142 | 2 949 |
| V_R_ | 1 659 423 | 4 453 879 | 21 | 2 609 | V_R_ | 1 657 946 | 5 345 371 | 21 | 3 058 |
| HZ_R_ | 8 682 999 | 4 039 938 | 143 | 2 397 | HZ_R_ | 8 652 633 | 4 761 988 | 142 | 2 767 |
| HZ_R+CU_ | 8 682 999 | 3 849 706 | 143 | 2 142 | HZ_R+CU_ | 8 652 633 | 4 539 164 | 142 | 2 461 |
| V_R_HZ_R_ | 1 659 423 | 4 119 191 | 21 | 2 459 | V_R_HZ_R_ | 1 657 946 | 4 889 634 | 21 | 2 864 |
| V_R_HZ_R+CU_ | 1 659 423 | 3 925 036 | 21 | 2 196 | V_R_HZ_R+CU_ | 1 657 946 | 4 661 616 | 21 | 2 544 |
| **TH=50** | | | | | | | | | |
| No Vacc. | 12 810 934 | 6 276 515 | 284 | 4 336 | No Vacc. | 12 764 884 | 8 040 539 | 282 | 5 541 |
| V_R_ | 1 669 663 | 6 316 848 | 22 | 4 502 | V_R_ | 1 667 756 | 8 446 503 | 22 | 5 995 |
| HZ_R_ | 12 810 934 | 5 619 596 | 284 | 3 689 | HZ_R_ | 12 764 884 | 7 087 872 | 282 | 4 623 |
| HZ_R+CU_ | 12 810 934 | 5 424 299 | 284 | 3 411 | HZ_R+CU_ | 12 764 884 | 6 860 259 | 282 | 4 291 |
| V_R_HZ_R_ | 1 669 663 | 5 620 812 | 22 | 3 819 | V_R_HZ_R_ | 1 667 756 | 7 373 353 | 22 | 4 964 |
| V_R_HZ_R+CU_ | 1 669 663 | 5 421 179 | 22 | 3 532 | V_R_HZ_R+CU_ | 1 667 756 | 7 140 358 | 22 | 4 617 |
| **TH=85** | | | | | | | | | |
| No Vacc. | 15 037 741 | 7 182 849 | 405 | 5 251 | No Vacc. | 14 979 582 | 9 470 327 | 400 | 7 052 |
| V_R_ | 1 677 687 | 6 889 018 | 25 | 5 346 | V_R_ | 1 674 796 | 9 468 512 | 24 | 7 423 |
| HZ_R_ | 15 037 741 | 6 375 958 | 405 | 4 335 | HZ_R_ | 14 979 582 | 8 259 078 | 400 | 5 689 |
| HZ_R+CU_ | 15 037 741 | 6 180 550 | 405 | 4 057 | HZ_R+CU_ | 14 979 582 | 8 031 516 | 400 | 5 357 |
| V_R_HZ_R_ | 1 677 687 | 6 063 818 | 25 | 4 412 | V_R_HZ_R_ | 1 674 796 | 8 145 008 | 24 | 5 966 |
| V_R_HZ_R+CU_ | 1 677 687 | 5 864 172 | 25 | 4 125 | V_R_HZ_R+CU_ | 1 674 796 | 7 912 017 | 24 | 5 620 |

**Table S4: Impact on the burden of illness, with 0% discount rate.** Cumulative number of infection cases and disease-related deaths, reported separately for varicella (natural and breakthrough) and HZ (with and without PHN), by vaccination policy, model and time horizon (TH=25, 50, 85). All outcomes are reported with a 0% discount rate for both benefits and costs.

| **Model TI** | | | | | **Model PI** | | | | |
| --- | --- | --- | --- | --- | --- | --- | --- | --- | --- |
| Policy | VAR cases | HZ cases | VAR deaths | HZ deaths |  | VAR cases | HZ cases | VAR deaths | HZ deaths |
| **TH=25** | | | | | | | | | |
| No Vacc. | 12 055 011 | 6 086 352 | 208 | 3 633 | No Vacc. | 12 007 234 | 7 354 137 | 207 | 4 268 |
| V_R_ | 1 754 138 | 6 228 546 | 23 | 3 745 | V_R_ | 1 750 614 | 7 609 322 | 23 | 4 450 |
| HZ_R_ | 12 055 011 | 5 580 023 | 208 | 3 384 | HZ_R_ | 12 007 234 | 6 672 324 | 207 | 3 951 |
| HZ_R+CU_ | 12 055 011 | 5 333 238 | 208 | 2 993 | HZ_R+CU_ | 12 007 234 | 6 386 367 | 207 | 3 478 |
| V_R_HZ_R_ | 1 754 138 | 5 697 257 | 23 | 3 485 | V_R_HZ_R_ | 1 750 614 | 6 881 878 | 23 | 4 111 |
| V_R_HZ_R+CU_ | 1 754 138 | 5 444 803 | 23 | 3 079 | V_R_HZ_R+CU_ | 1 750 614 | 6 588 076 | 23 | 3 616 |
| **TH=50** | | | | | | | | | |
| No Vacc. | 24 058 794 | 11 605 260 | 638 | 8 955 | No Vacc. | 23 974 472 | 15 641 082 | 632 | 12 078 |
| V_R_ | 1 785 205 | 11 535 623 | 27 | 9 348 | V_R_ | 1 780 114 | 16 619 849 | 26 | 13 335 |
| HZ_R_ | 24 058 794 | 10 126 258 | 638 | 7 194 | HZ_R_ | 23 974 472 | 13 428 136 | 632 | 9 521 |
| HZ_R+CU_ | 24 058 794 | 9 868 173 | 638 | 6 750 | HZ_R+CU_ | 23 974 472 | 13 131 713 | 632 | 8 991 |
| V_R_HZ_R_ | 1 785 205 | 9 962 363 | 27 | 7 490 | V_R_HZ_R_ | 1 780 114 | 14 075 678 | 26 | 10 439 |
| V_R_HZ_R+CU_ | 1 785 205 | 9 697 648 | 27 | 7 030 | V_R_HZ_R+CU_ | 1 780 114 | 13 770 899 | 26 | 9 884 |
| **TH=85** | | | | | | | | | |
| No Vacc. | 39 407 227 | 17 886 029 | 1 476 | 15 266 | No Vacc. | 39 249 090 | 25 512 560 | 1 454 | 22 443 |
| V_R_ | 1 840 706 | 15 235 455 | 45 | 14 959 | V_R_ | 1 828 869 | 22 966 934 | 42 | 22 502 |
| HZ_R_ | 39 407 227 | 15 369 418 | 1 476 | 11 652 | HZ_R_ | 39 249 090 | 21 516 282 | 1 454 | 16 832 |
| HZ_R+CU_ | 39 407 227 | 15 110 796 | 1 476 | 11 208 | HZ_R+CU_ | 39 249 090 | 21 220 199 | 1 454 | 16 304 |
| V_R_HZ_R_ | 1 840 706 | 12 827 694 | 45 | 11 432 | V_R_HZ_R_ | 1 828 869 | 18 844 624 | 42 | 16 875 |
| V_R_HZ_R+CU_ | 1 840 706 | 12 562 902 | 45 | 10 972 | V_R_HZ_R+CU_ | 1 828 869 | 18 539 978 | 42 | 16 320 |

**Table S5: Economic and QoL impact, with discount rate at 0%.** Total cost (accounting for cost of disease and cost of policy, in million EUR), total QALY loss (in thousands), ICER, and NMB (in million EUR), based on both the marginal productivity of the national health system (NMB_k_, t=15 000 EUR) and on the consumption value of health (NMB_v_, t=40 000 EUR), by policy, model, and time horizon. All NMBs are calculated with respect to the no vaccination. All outcomes are reported with a 0% discount rate for both benefits and costs. The policies are sorted in ascending order by their total cost.

| **Model TI** | | | | | | **Model PI** | | | | | |
| --- | --- | --- | --- | --- | --- | --- | --- | --- | --- | --- | --- |
| **Policy** | **Total cost** | **QALY loss** | **ICER*** | **NMBk** | **NMBv** | **Policy** | **Total cost** | **QALY loss** | **ICER*** | **NMBk** | **NMBv** |
| *Time Horizon = 25 years* | | | | | | | | | | | |
| No Vacc. | 2 532 | 649 |  |  |  | No Vacc. | 2 855 | 777 |  |  |  |
| V_R_ | 2 547 | 637 | 1 171 | 176 | 494 | V_R_ | 2 911 | 779 | SD | -87 | -137 |
| HZ_R_ | 3 550 | 580 | WD | 16 | 1 741 | HZ_R_ | 3 826 | 685 | WD | 405 | 2 699 |
| HZ_R+CU_ | 3 557 | 564 | WD | 251 | 2 378 | HZ_R+CU_ | 3 869 | 681 | WD | 424 | 2 821 |
| V_R_HZ_R_ | 3 807 | 537 | WD | 413 | 3 225 | V_R_HZ_R_ | 4 069 | 635 | WD | 915 | 4 464 |
| V_R_HZ_R+CU_ | 3 811 | 520 | 10 812 | 666 | 3 908 | V_R_HZ_R+CU_ | 4 109 | 629 | 8 494 | 961 | 4 652 |
| *Time Horizon = 50 years* | | | | | | | | | | | |
| No Vacc. | 5 008 | 1 339 |  | - | - | No Vacc. | 6 129 | 1 806 |  | - | - |
| V_R_ | 4 881 | 1 313 | cost-saving | 509 | 1 145 | V_R_ | 6 338 | 1 925 | SD | -2 004 | -4 996 |
| V_R_HZ_R_ | 6 436 | 1 069 | WD | 2 625 | 9 379 | V_R_HZ_R_ | 7 488 | 1 466 | 3 995 | 3 743 | 12 247 |
| HZ_R_ | 6 594 | 1 108 | SD | 1 870 | 7 630 | HZ_R_ | 7 592 | 1 536 | SD | 2 588 | 9 341 |
| V_R_HZ_R+CU_ | 6 681 | 1 022 | 5 269 | 3 077 | 10 994 | V_R_HZ_R+CU_ | 7 721 | 1 414 | 4 507 | 4 287 | 14 087 |
| HZ_R+CU_ | 6 842 | 1 063 | SD | 2 301 | 9 192 | HZ_R+CU_ | 7 823 | 1 482 | SD | 3 160 | 11 249 |
| *Time Horizon = 85 years* | | | | | | | | | | | |
| No Vacc. | 7 937 | 2 093 |  | - | - | No Vacc. | 10 119 | 3 018 |  | - | - |
| V_R_ | 7 012 | 1 834 | cost-saving | 4 819 | 11 308 | V_R_ | 9 337 | 2 866 | cost-saving | 3 059 | 6 854 |
| V_R_HZ_R_ | 9 287 | 1 447 | WD | 8 346 | 24 505 | V_R_HZ_R_ | 11 065 | 2 214 | 2 652 | 11 105 | 31 191 |
| V_R_HZ_R+CU_ | 9 559 | 1 400 | 5 879 | 8 771 | 26 092 | V_R_HZ_R+CU_ | 11 323 | 2 161 | 4 822 | 11 649 | 33 071 |
| HZ_R_ | 10 179 | 1 691 | SD | 3 787 | 13 834 | HZ_R_ | 11 886 | 2 387 | SD | 7 689 | 23 449 |
| HZ_R+CU_ | 10 454 | 1 646 | SD | 4 192 | 15 373 | HZ_R+CU_ | 12 148 | 2 336 | SD | 8 204 | 25 259 |

**^*^**SD, strong dominance (a policy is dominated when the alternative is less costly and more effective); WD, weak dominance (a policy is dominated when its ICER is larger than that of a policy with higher effectiveness).

### Probabilistic Sensitivity Analysis

### *Additional results on the PSA for the main analysis*

The robustness of model results to uncertainty in model parameters is assessed through a probabilistic sensitivity analysis (PSA), performed using a Bayesian approach, whereby all parameters are varied simultaneously sampling from the probability distributions specified in Table 1 of the main text. Results are shown in Fig. S13 where the boxplots of the NMB are shown for the five considered strategies, for the two models (TI and PI), and for the three different time horizons (25, 50 and 85 years).

**Figure S13:** **PSA for baseline scenario (varicella vaccination coverage at 80%).** Box plots of the posterior distribution of NMB_k_ (with 2.5%, 25%, 50%, 75%, and 97.5% quantiles), obtained from the comparison between each policy and the no intervention scenario using the conservative supply-based threshold (15 000 EUR), under model TI (top row) and model PI (bottom row), under each time-horizon (from left to right: 25 years, 50 years, 85 years). Discount rate for both benefits and costs is equal to 3%.

For each strategy, we also report the probability of being optimal (i.e. generating the highest NMB) per different levels of the CE threshold, using CE acceptability curves [27], for both discount rates of 0% and 3% (Fig. S14).

Under model TI, we see that, for very low thresholds (up to 6,000 or 8,000 EUR, depending on the discount rate), the policy V_R_ is the most cost-effective one. After that threshold, the policy V_R_HZ_R+CU_ is always the most cost-effective one, under model TI. Instead, for model PI, we see that the policy V_R_ is the most cost-effective up to a threshold of 5,000 EUR, then it is briefly replaced, under discount rate of 0%, by policy V_R_HZ_R_ up to 7,000 EUR, to be finally replaced by policy V_R_HZ_R+CU_ (under discount rate of 0%), while, under discount rate of 3%, it is replaced by a combination of HZ_R+CU_ (with a probability of around 40% of being optimal) and V_R_HZ_R+CU_ (with a probability of around 60% of being optimal).

**Figure S14: CEAC vs. no vaccination.** The CEACs show the probability of each strategy to be the most cost-effective one for different values of the cost-effectiveness (CE) threshold. Results are shown for both model TI (panels A and C) and PI (panels B and D) under the longest time horizon (85 years). Discount rates for both costs and benefits are assumed equal to 0% (panels A and B) and to 3% (panels C and D).

### *Sensitivity Analysis*

### Low and high varicella vaccination coverage levels

Two alternative scenarios are considered here to assess the sensitivity of our results to different coverage levels of varicella vaccination (70%, 95%). We notice that a lower varicella vaccination (scenario #1, Fig. S15) coverage produces higher NMB, as it does not fully reduce virus circulation and boosting, and thus producing a smaller increase in HZ incidence (which is the one leading to higher costs of disease). On the other hand, a higher varicella vaccination coverage (scenario #2, Fig. S16) completely reduces with time the virus circulation and, consequently, the boosting effect. It follows that, in the short and in the medium run, we see a larger increase in HZ incidence and thus in the costs of disease, which in turn leads to much lower NMB.

**Figure S15:** **PSA for sensitivity scenario #1 (varicella vaccination coverage at 70%).** Box plots of the posterior distribution of NMB_k_ (with 2.5%, 25%, 50%, 75%, and 97.5% quantiles), obtained from the comparison between each policy and the no intervention scenario using the supply-based threshold, under model TI (top row) and model PI (bottom row), under each time horizon (from left to right: 25 years, 50 years, 85 years). Discount rate for both benefits and costs is equal to 3%.

**Figure S16: PSA for sensitivity scenario #2 (varicella vaccination coverage at 95%).** Box plots of the posterior distribution of NMBk (with 2.5%, 25%, 50%, 75%, and 97.5% quantiles), obtained from the comparison between each policy and the no intervention scenario using the supply-based threshold, under model TI (top row) and model PI (bottom row), under each time horizon (from left to right: 25 years, 50 years, 85 years). Discount rate for both benefits and costs is equal to 3%.

### Extreme scenarios on the boosting effect ($\boldsymbol{\chi}\mathbf{,}\boldsymbol{z}\mathbf{)}$

In order to evaluate the robustness of model outcomes to the indirect effects of varicella vaccination on HZ, we investigate two extreme scenarios representative of two almost opposite assumptions on the role of exogenous boosting in shaping HZ epidemiology. The first one represents a *worst-case* scenario (scenario #3, Fig. S17) for the potential effects of varicella vaccination on HZ as we extracted a sub sample of model runs generated with a high reactivation rate among vaccinated individuals (χ=0.23), and a high force of boosting (*z* values in the upper 10% of the distribution). On the opposite, a *best-case* scenario (scenario #4, Fig. S18) is considered extracting those runs with a low reactivation rate (χ=0.08), and a low force of boosting (*z* values in the lower 10% of the distribution).

**Figure S17: PSA for sensitivity scenario #3 (high** $\boldsymbol{z}$**, high** $\boldsymbol{\chi}$**, worst case scenario for HZ).** Box plots of the posterior distribution of NMB_k_ (with 2.5%, 25%, 50%, 75%, and 97.5% quantiles), obtained from the comparison between each policy and the no intervention scenario using the supply-based threshold, under model TI (top row) and model PI (bottom row), under each time horizon (from left to right: 25 years, 50 years, 85 years). Discount rate for both benefits and costs is equal to 3%.

**Figure S18:** **PSA for sensitivity scenario #4 (low** $\boldsymbol{z}$**, low** $\boldsymbol{\chi}$**, best case scenario for HZ).** Box plots of the posterior distribution of NMB_k_ (with 2.5%, 25%, 50%, 75%, and 97.5% quantiles), obtained from the comparison between each policy and the no intervention scenario using the supply-based threshold, under model TI (top row) and model PI (bottom row), under each time horizon (from left to right: 25 years, 50 years, 85 years). Discount rate for both benefits and costs is equal to 3%.

### Demographic sensitivity analysis

### The analysis presented in the main text is based on demographic projections for the period 2016-2100 associated with the “medium” fertility scenario, as reported in the 2015 World Population Prospects (WPP) [3]. This scenario shows a rather stable birth rate after 2016 (Figure S1). In order to assess the sensitivity of our results to the uncertainty surrounding future demographic changes, we simulated the same analysis presented in the main text by assuming two alternative scenarios for the future birth rate provided in the 2015 WPP: the “low” and “high” fertility scenarios shown in Fig S1. The future evolution of the age distribution of the Italian population under the three demographic projections reported in the WPP is shown in Fig S19 along with the age distribution of the population simulated by our models. Please note that the demographic component of models PI and TI is the same.

###
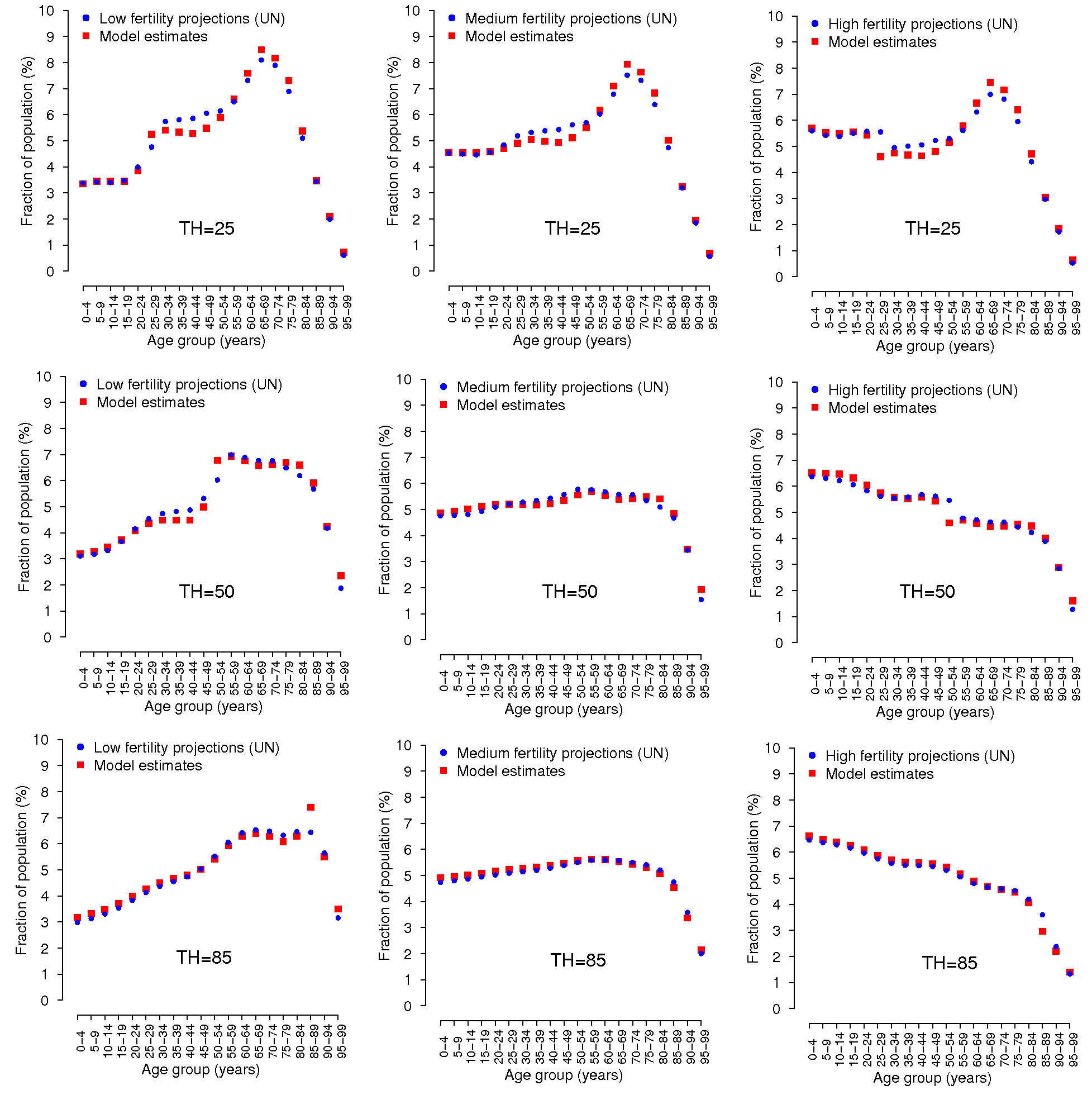
Figure S19: Age distribution of the Italian population as reported in the 2015 UN World Population Prospects (blue) and as obtained in our demographic model (red). Different rows correspond to different time horizons (25 years, 50 years, 85 years). The first column shows the expected evolution of the age structure of the Italian population under the “low” fertility scenario, the second column the “medium” fertility and the third column to the “high” fertiliy scenario.

### We found that the progressive decrease in the fraction of children expected in the Italian population under the low fertility scenario (left column, Figure S19) would result in a lower circulation of varicella in the next decades and, in turn, in a lower level of boosting compared with results obtained under the medium fertility scenario (see Figure S20A). As a consequence, in this demographic scenario, both models estimate a higher incidence of HZ under all vaccination strategies considered, compared to what obtained with the medium fertility scenario (Fig S20B-D and Fig S22 top row).

A))


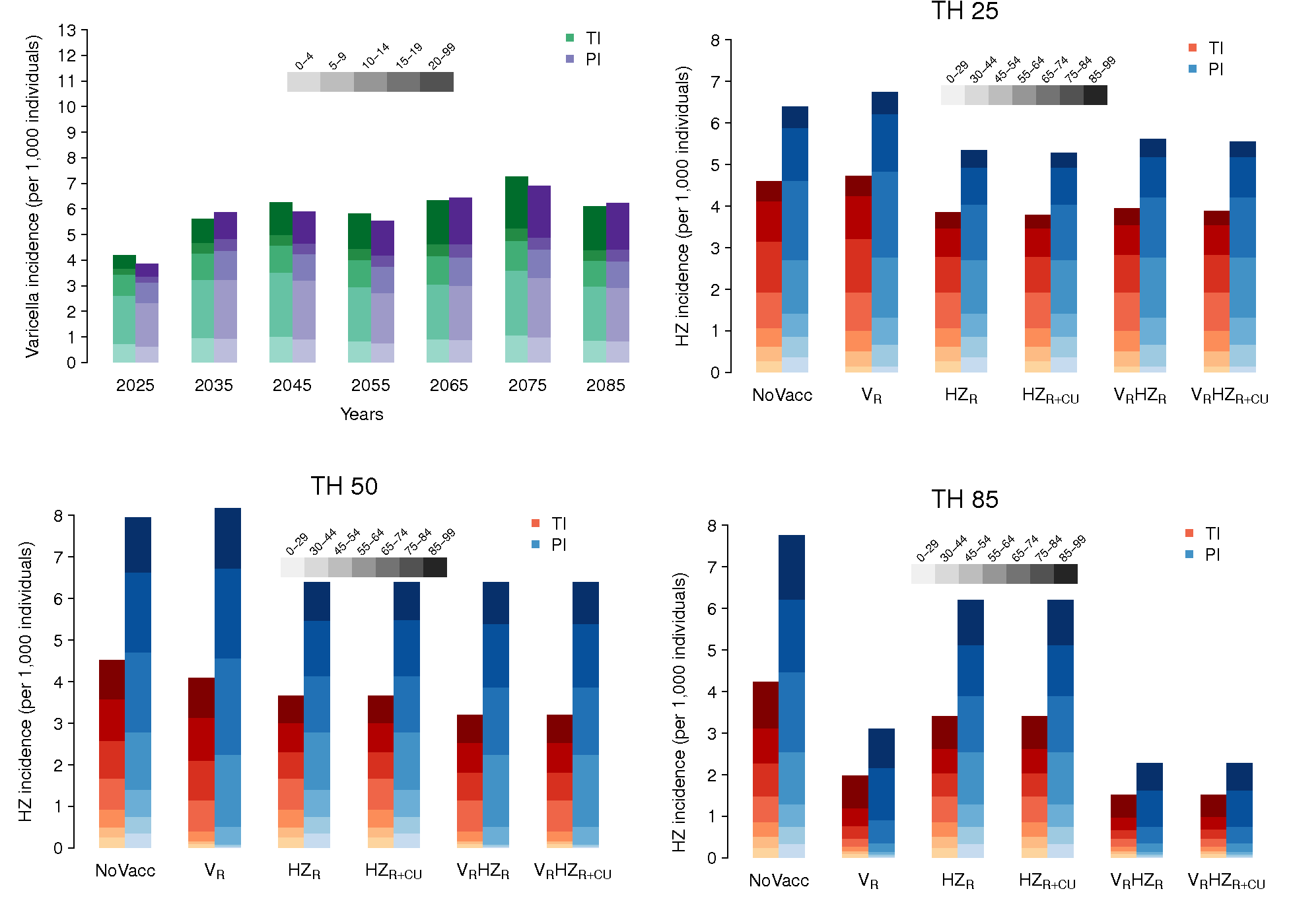


**Figure S20: Varicella and HZ by age under the low fertility scenario for demographic projections**: A) Estimated total varicella incidence over time, stratified by age groups, as obtained by model TI and PI, under the no vaccination scenario. B)-D) Estimated total HZ incidence, stratified by age groups, as obtained by model TI and PI, under the different considered policies at a time horizon (TH) of 25 years, 50 years, and 85 years.

B))

C))

D

))

### Conversely, the increase in the fraction of children expected in the high fertility scenario (right column, Figure S19) results in a higher circulation of varicella and in a higher level of boosting compared with results obtained under the medium fertility scenario (see Figure S21A). As a consequence, the incidence of HZ expected under the “high” fertility scenario is lower than the one estimated under the medium fertility scenario (Figure S21B-D and S22 bottom row), for all the vaccination strategies considered. Remarkably, obtained results confirm that the incidence of HZ is much less sensitive to changes in varicella circulation in model TI than in model PI (Figure S22).


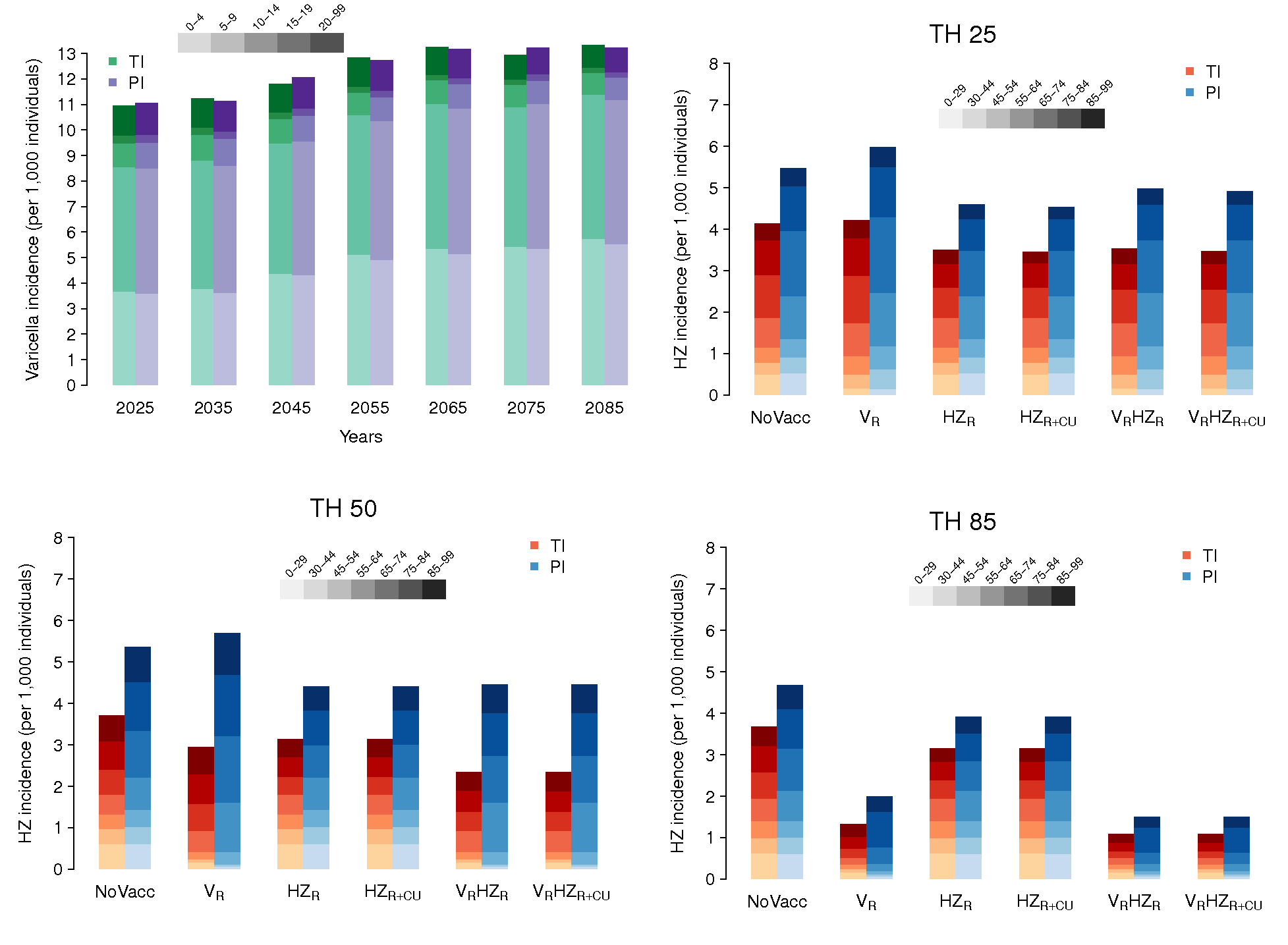


**Figure S21: Varicella and HZ by age under the high fertility scenario for demographic projections**: A) Estimated total varicella incidence over time, stratified by age groups, as obtained by model TI and PI, under the no vaccination scenario. B)-D) Estimated total HZ incidence, stratified by age groups, as obtained by model TI and PI, under the different considered policies at a time horizon (TH) of 25 years, 50 years, and 85 years.

A))

B

))

C

D))

**Figure S22:** Total HZ incidence over time under the different vaccination strategies as estimated by A) Model TI assuming the “low” fertility scenario for demographic projections B) Model PI assuming the “low” fertility scenario. C) Model TI assuming the “high” fertility scenario for demographic projections D) Model PI assuming the “high” fertility scenario.


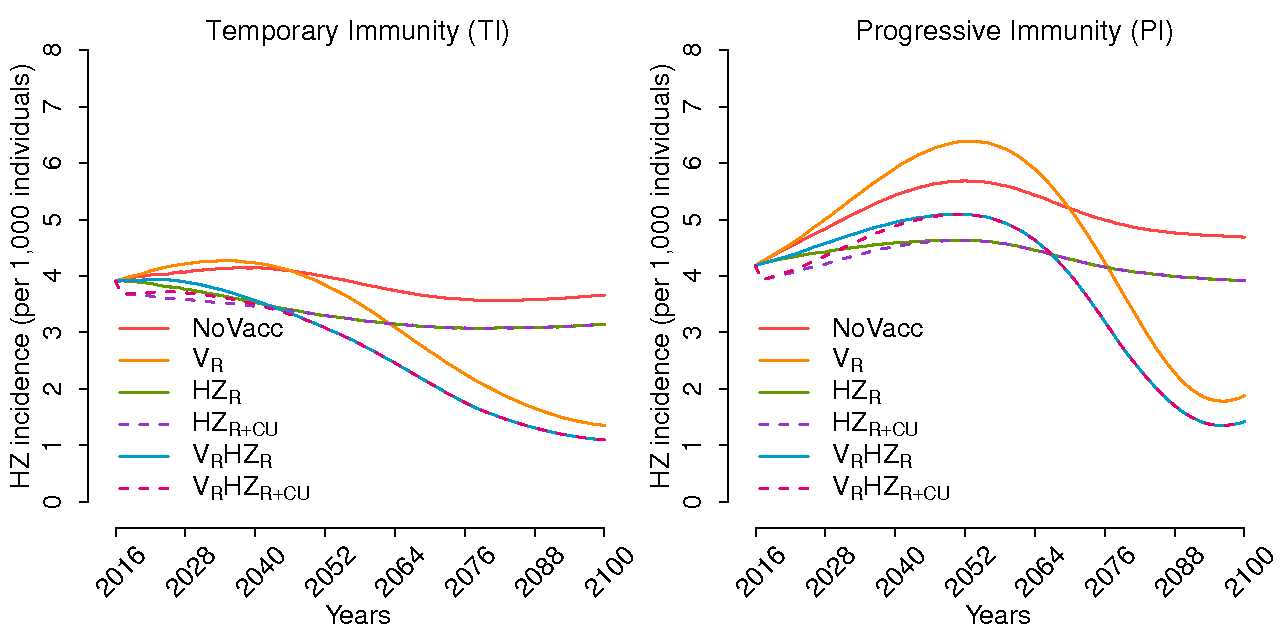

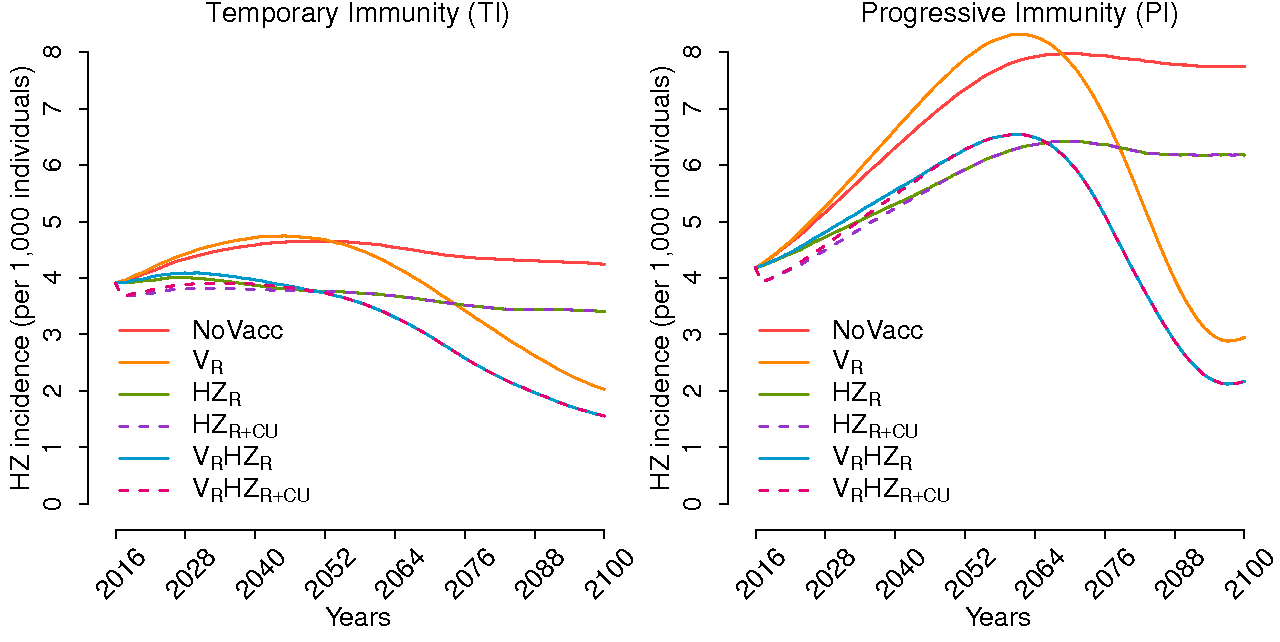


D))

C))

B))

A))

Note that different assumptions on the future birth rate correspond to different estimates for the population size (growing population under the “high” fertility scenario, decreasing population under the “low” fertility scenario). Thus, even though the incidence of HZ estimated under the “high” birth rate scenario is lower than the one estimated under the “low” fertility scenario, the former can mask a higher absolute number of HZ cases compared to the latter.

In the two graphs below, we show the box plots of the NMB for the considered programmes (with k=15,000, discount rate 3%, time horizon 85 years) under the low birth rate scenario (Fig. S23, left panel for model TI and right panel for model PI) and under the high birth rate (Fig. S24 left panel for model TI and right panel for model PI).

Under model TI, where the exogenous boosting is present, but weaker than under model PI, the strategies containing varicella vaccination (V_R_, V_R_HZ_R_, V_R_HZ_R+CU_) generate higher NMBs as the birth rate increases (because more varicella cases are averted). Conversely, under the strategies based only on HZ vaccination (HZ_R_, HZ_R+CU_), the NMBs slightly decrease as a consequence of the overall final increase in the number of HZ cases under the “high” fertility scenario.

Similarly, under model PI, we found that all strategies generated slightly lower NMB under the “high” fertility scenario than under the “low” fertility one, because of the higher number of HZ cases estimated under the former scenario.

**Figure S23:** **PSA for sensitivity scenario #5 (lower birth rate).** Box plots of the posterior distribution of NMB_k_ (with 2.5%, 25%, 50%, 75%, and 97.5% quantiles), obtained from the comparison between each policy and the no intervention scenario using the supply-based threshold, under model TI (left panel) and model PI (right panel), under the longest time horizon (85 years). Discount rate for both benefits and costs is equal to 3%.

**Figure S24:** **PSA for sensitivity scenario #6 (higher birth rate).** Box plots of the posterior distribution of NMB_k_ (with 2.5%, 25%, 50%, 75%, and 97.5% quantiles), obtained from the comparison between each policy and the no intervention scenario using the supply-based threshold, under model TI (left panel) and model PI (right panel), under the longest time horizon (85 years). Discount rate for both benefits and costs is equal to 3%.

### Assessing the impact of the different sources of uncertainty

### *Univariate sensitivity analysis*

The impact of the uncertainty relative to each parameter, considered singularly, on the overall cost-effectiveness of a policy is evaluated for the most cost-effective policy (V_R_HZ_R+CU_) against the no vaccination scenario, and considering the longest time horizon (TH=85) (Fig. S25). Results show that, no matter the model TI or PI used, the parameters having the largest effect on the ICER are those related to HZ and PHN, such as the outpatient cost for HZ (including visit and treatment), the QALY loss for HZ, and the hospitalization costs for HZ. Conversely, the QALY loss for natural varicella and for breakthrough varicella have a much smaller impact.

**Figure S25:** **Univariate sensitivity analysis.** Tornado charts reporting the effect of the uncertainty of each parameter, taken singularly, on the ICER for the comparison between the most cost-effective policy (V_R_HZ_R+CU_) and the no vaccination scenario. The uncertainty is given by the values corresponding to the lower and upper bound of the 95% credible intervals predicted by the PSA. Results are obtained under model TI (top panel) and PI (bottom panel), for the longest time horizon (85 years). The discount rate for both benefits and costs is equal to 3%.

### *PSA – Epidemiological vs. economic uncertainty*

To assess which source of uncertainty is more influential with respect to the NMB, we compared the uncertainty deriving only from the transmission model for the epidemiological outcomes when keeping the economic parameters fixed (i), the one deriving only from the input parameters of the economic model when keeping the epidemiological parameters fixed (ii), and both sources of uncertainty together. For this purpose, we show the box plots of the posterior distribution of the NMB (Fig. S26), and we calculated a robust statistical measure of relative dispersion, i.e. the coefficient of quartile variation (CQD), derived as $\left( Q_{3}-Q_{1} \right)/{|\left( Q_{3}+Q_{1} \right)|}$, where $Q_{3}$ and $Q_{1}$ are the 25% and the 75% quantiles, respectively (Table S6). We note that the most influential source of uncertainty is the one around the epidemiological outcomes, and it appears to be much larger than the one around the economic parameters. Indeed, if we consider the two sources of uncertainty together, we see that both the box plots (third column for each strategy) and the CQDs are quite similar to those relative to the epidemiological parameters only, and are both larger than those relative to the economic parameters only.

**Figure S26: Comparison among the different sources of uncertainty around the NMB.** Box plots of the posterior distribution of NMBk (with 2.5%, 25%, 50%, 75%, and 97.5% quantiles), obtained from the comparison between each policy and the no intervention scenario using the conservative supply-based threshold (15,000 EUR), under model PI (left panel) and model TI (right panel), under the longest time-horizon (85 years). Discount rate for both benefits and costs is equal to 3%. For each policy, we show the uncertainty around the output from the IBM model for the epidemiological outcomes when keeping the economic parameters fixed (EPI: green box plot), the uncertainty around the input parameters for the economic model when keeping the epidemiological parameters fixed (ECO: red box plot), and both sources of uncertainty together (EPI-ECO: purple box plot).

**Table S6: Comparison among the different sources of uncertainty around the NMB.** Estimates of the coefficient of quartile deviation (CQD) of the posterior distribution of NMBk, obtained from the comparison between each policy and the no intervention scenario using the conservative supply-based threshold (15000 EUR), under model TI and model PI), under the longest time-horizon (85 years). Discount rate for both benefits and costs is equal to 3%. For each policy, we show the CQD related to the uncertainty around the output from the transmission model for the epidemiological outcomes when keeping the economic parameters fixed (EPI), the uncertainty around the input parameters for the economic model when keeping the epidemiological parameters fixed (ECO) and both sources of uncertainty together (EPI-ECO).

|  | TI | | | PI | | |
| --- | --- | --- | --- | --- | --- | --- |
|  | EPI | ECO | EPI-ECO | EPI | ECO | EPI-ECO |
| V_R_ | 0.77 | 0.16 | 0.76 | 1.29 | 0.61 | 1.40 |
| HZ_R_ | 0.32 | 0.12 | 0.36 | 0.22 | 0.079 | 0.23 |
| HZ_R+CU_ | 0.33 | 0.12 | 0.37 | 0.23 | 0.080 | 0.25 |
| V_R_ HZ_R_ | 0.24 | 0.091 | 0.26 | 0.39 | 0.082 | 0.41 |
| V_R_ HZ_R+CU_ | 0.22 | 0.090 | 0.24 | 0.37 | 0.080 | 0.39 |

### *Changing the status quo strategy: HZ vaccination as reference*

To mimic those countries (such as the UK) that have already introduced HZ vaccination and are now considering the possibility to add varicella vaccination on top of the elderly programme, we also considered a different scenario, where the status quo is the 1-dose HZ vaccination at 65 years of age. We compared two possible strategies: replacing the elderly HZ vaccination with the childhood varicella vaccination, or having both of them together. Considering the long term (TH=85) and a discount rate of 3%, we found that it is always cost-effective to have a combined strategy with varicella childhood vaccination and HZ vaccination of the elderly, no matter the chosen exogenous boosting assumption (model TI or PI). Conversely, the replacement of the elderly HZ vaccination with the varicella childhood vaccination would likely be cost-ineffective under the PI assumption (Fig. S27).

**Figure S27: PSA with HZ_R_ as status quo:** Box plots of the posterior distribution of NMB_k_ (with 2.5%, 25%, 50%, 75%, and 97.5% quantiles), obtained from the comparison between each policy and the strategy HZ_R_ using the supply-based threshold, under model TI (left panel) and model PI (right panel), under the longest time horizon (85 years). Discount rate for both benefits and costs is equal to 3%.

### References

1. Human Mortality Database. University of California, Berkeley (USA), and Max Planck Institute for Demographic Research (Germany). http:// [www.mortality.org](http://www.mortality.org/). Accessed on 3 May 2018.

2. Database of the Italian Institute of Statistics. Italian National Institute of Statistics (ISTAT), Rome, Italy. http://demo.istat.it/. Accessed on 3 May 2018.

3. United Nations, Department of Economic and Social Affairs, Population Division. World Population Prospects: The 2015 Revision, Key Findings and Advanced Tables. Working Paper. 2015.

4. Fumanelli L, Ajelli M, Manfredi P, Vespignani A. Inferring the structure of social contacts from demographic data in the analysis of infectious diseases spread. PLoS Comput Biol. 2012.

5. Gabutti G, Penna C, Rossi M, Salmaso S, Rota MC, Bella A, et al. The seroepidemiology of varicella in Italy. Epidemiol Infect. 2001;126:433–40.

6. Gialloreti LE, Merito M, Pezzotti P, Naldi L, Gatti A, Beillat M, et al. Epidemiology and economic burden of herpes zoster and post-herpetic neuralgia in Italy: a retrospective, population-based study. BMC Infect Dis. 2010;10:230–11.

7. Hope-Simpson RE. The nature of herpes zoster: a long-term study and a new hypothesis. Proc R Soc Med. 1965;58:9–20.

8. Guzzetta G, Poletti P, Del Fava E, Ajelli M, Scalia Tomba G, Merler S, et al. Hope-Simpson's progressive immunity hypothesis as a possible explanation for herpes zoster incidence data. Am J Epidemiol. 2013;177:1134–42.

9. Guzzetta G, Poletti P, Merler S, Manfredi P. The Epidemiology of Herpes Zoster After Varicella Immunization Under Different Biological Hypotheses: Perspectives From Mathematical Modeling. Am J Epidemiol. 2016;183:765–73.

10. Brisson M, Edmunds WJ, Gay NJ, Law B, De Serres G. Modelling the impact of immunization on the epidemiology of varicella zoster virus. Epidemiol Infect. 2000;125:651–69.

11. Brisson M, Melkonyan G, Drolet M, De Serres G, Thibeault R, De Wals P. Modeling the impact of one- and two-dose varicella vaccination on the epidemiology of varicella and zoster. Vaccine. 2010;28:3385–97.

12. van Hoek AJ, Melegaro A, Gay NJ, Bilcke J, Edmunds WJ. The cost-effectiveness of varicella and combined varicella and herpes zoster vaccination programmes in the United Kingdom. Vaccine. 2012;30:1225–34.

13. Marziano V, Poletti P, Guzzetta G, Ajelli M, Manfredi P, Merler S. The impact of demographic changes on the epidemiology of herpes zoster: Spain as a case study. Proc R Soc B. 2015;282:20142509–9.

14. Seward JF, Zhang JX, Maupin TJ, Mascola L, Jumaan AO. Contagiousness of Varicella in Vaccinated Cases: A Household Contact Study. J Am Med Assoc. 2004;292:704–8.

15. Manfredi P, Williams JR. Realistic population dynamics in epidemiological models: the impact of population decline on the dynamics of childhood infectious diseases: Measles in Italy as an example. Math Biosci. 2004;192:153–75.

16. van Hoek AJ, Melegaro A, Zagheni E, Edmunds WJ, Gay NJ. Modelling the impact of a combined varicella and zoster vaccination programme on the epidemiology of varicella zoster virus in England. Vaccine. 2011;29:2411–20.

17. Poletti P, Melegaro A, Ajelli M, Del Fava E, Guzzetta G, Faustini L, et al. Perspectives on the Impact of Varicella Immunization on Herpes Zoster. A Model-Based Evaluation from Three European Countries. PLoS ONE. 2013;8:e60732.

18. Gilks WR, Richardson S, Spiegelhalter D. Markov Chain Monte Carlo in Practice. CRC Press; 1995.

19. Civen R, Chaves SS, Jumaan AO, Wu H, Mascola L, Gargiullo PM, et al. The incidence and clinical characteristics of herpes zoster among children and adolescents after implementation of varicella vaccination. Pediatr Infect Dis J. 2009;28:954–9.

20. Benenson AS. Control of communicable diseases manual. American Public Health Association; 1995.

21. Vázquez M, LaRussa PS, Gershon AA, Steinberg SP, Freudigman K, Shapiro ED. The Effectiveness of the Varicella Vaccine in Clinical Practice. N Engl J Med. 2001;344:955–60.

22. Oxman MN, Levin MJ, Johnson GR, Schmader KE, Straus SE, Gelb LD, et al. A Vaccine to Prevent Herpes Zoster and Postherpetic Neuralgia in Older Adults. N Engl J Med. 2009;352:2271–84.

23. van Hoek AJ, Ngama M, Ismail A, Chuma J, Cheburet S, Mutonga D, et al. A Cost Effectiveness and Capacity Analysis for the Introduction of Universal Rotavirus Vaccination in Kenya: Comparison between Rotarix and RotaTeq Vaccines. PLoS ONE. 2012;7:e47511–10.

24. Brisson M, Edmunds WJ. Varicella vaccination in England and Wales: Cost-utility analysis. Arch Dis Child. 2003;88:862–9.

25. Chaves SS, Zhang J, Civen R, Watson BM, Carbajal T, Perella D, et al. Varicella disease among vaccinated persons: clinical and epidemiological characteristics, 1997-2005. J Infect Dis. 2008;197 Suppl 2:S127–31.

26. Thiry N, Beutels P, Van Damme P, Van Doorslaer E. Economic Evaluations of Varicella Vaccination Programmes. Pharmacoeconomics. 2003;21:13–38.

27. Fenwick E, Claxton K, Sculpher M. Representing uncertainty: the role of cost-effectiveness acceptability curves. Health Econ. 2001;10:779–87.
